# Microbiological and Chemical Quality of Packaged Sachet Water and Household Stored Drinking Water in Freetown, Sierra Leone

**DOI:** 10.1371/journal.pone.0131772

**Published:** 2015-07-10

**Authors:** Michael B. Fisher, Ashley R. Williams, Mohamed F. Jalloh, George Saquee, Robert E. S. Bain, Jamie K. Bartram

**Affiliations:** 1 The Water Institute at UNC, Department of Environmental Sciences and Engineering, University of North Carolina, Chapel Hill, North Carolina, United States of America; 2 FOCUS 1000, Freetown, Sierra Leone; Catalan Institute for Water Research (ICRA), SPAIN

## Abstract

Packaged drinking water (PW) sold in bottles and plastic bags/sachets is widely consumed in low- and middle-income countries (LMICs), and many urban users in sub-Saharan Africa (SSA) rely on packaged sachet water (PSW) as their primary source of water for consumption. However, few rigorous studies have investigated PSW quality in SSA, and none have compared PSW to stored household water for consumption (HWC). A clearer understanding of PSW quality in the context of alternative sources is needed to inform policy and regulation. As elsewhere in SSA, PSW is widely consumed in Sierra Leone, but government oversight is nearly nonexistent. This study examined the microbiological and chemical quality of a representative sample of PSW products in Freetown, Sierra Leone at packaged water manufacturing facilities (PWMFs) and at points of sale (POSs). Samples of HWC were also analyzed for comparison. The study did not find evidence of serious chemical contamination among the parameters studied. However, 19% of 45 PSW products sampled at the PWMF contained detectable *Escherichia coli* (EC), although only two samples exceeded 10 CFU/100 mL. Concentrations of total coliforms (TC) in PSW (but not EC) increased along the supply chain. Samples of HWC from 60 households in Freetown were significantly more likely to contain EC and TC than PSW at the point of production (p<0.01), and had significantly higher concentrations of both bacterial indicators (p<0.01). These results highlight the need for additional PSW regulation and surveillance, while demonstrating the need to prioritize the safety of HWC. At present, PSW may be the least unsafe option for many households.

## Introduction

Access to safe drinking water is critical to human health and development [1]. Efforts to ensure access to safe drinking water have historically focused on communal drinking water sources and piped supplies. However, the consumption of packaged water (PW) has grown rapidly in recent decades, first in high-income countries (HICs), and more recently in low and middle-income countries (LMICs). PW is drinking water packaged in plastic or glass bottles, sachets, or bags in a range of sizes, and may be sold in shops, on the street, or delivered to homes. The scale of PW consumption is substantial: in 2011, documented global bottled water (BW) sales exceeded 225 billion liters [2]. While statistics on sales of packaged sachet water (PSW, drinking water packaged in sealed plastic sleeves, typically 500-mL) are more difficult to obtain, consumption is increasing rapidly—especially in LMICs [3]. According to the 2011 Multiple Indicator Cluster Survey for Ghana, 18.3% of households listed PSW as their main source of water for consumption, 5.4% of rural households and 32.2% of urban households [4]. A recent study in a peri-urban area of Accra found that 47% of respondents listed PSW as their primary source of water for consumption [5]. The volumes of PSW consumed are often substantial relative to total daily drinking water consumption: a study of 137 PW users in Ibadan, Nigeria found that 58% consumed between two and four 500-mL sachets per day, while 28% consumed more than four sachets per day [6].

While PSW consumption is growing rapidly in LMICs, many are unable to effectively regulate the safety of PSW products sold within their borders [7, 8]. Some PSW manufacturers voluntarily adhere to quality, safety, and hygiene standards, but others lack the capacity or will to do so in the absence of effective regulation, monitoring, and/or enforcement. There is thus significant concern among governments and international organizations that inadequately regulated PSW may pose serious health risks [9]. Recently, concerns have been raised about possible links of PSW to outbreaks of cholera [10] and other waterborne diseases. There is also concern about the impact of PSW production on overburdened municipal water supplies, as well as the environmental consequences of improperly managed plastic waste from PSW products [5]; these issues, while important, are outside the scope of the current study.

Studies have shown that PSW products in West African countries are prone to microbiological [10–13], chemical [14–16] and radiological [17] contamination. However, the risks related to PSW consumption must be weighed against those of consuming water from alternative sources. In many LMICs, safe, continuous, at-home water supply is uncommon, and most households collect water from off-premises sources, storing it under conditions that make subsequent contamination probable [18, 19]. Even when safe piped supplies are available, intermittent service can cause households to adopt coping strategies that may result in contamination through unsafe water storage practices and use of unprotected sources [20]. Piped and other improved drinking water sources outside the home are often unsafe as well [21]. Thus, it is important to consider the health implications of PSW quality in the context of alternative drinking water options.

In Sierra Leone (SL), as in many other West African countries, PSW consumption is extremely prevalent, particularly in the capital city of Freetown, and is increasing in other cities. The PSW market in Sierra Leone includes large producers based primarily in Freetown, as well as smaller producers both within and outside of the capital. Most smaller operations consist of an imported “package plant,” comprising a water treatment and sachet filling system located in a home or shed, and generally using municipal piped water as the raw water source. While treatment methods vary widely, a nationwide survey of 77 PSW and BW manufacturers found that all reported some form of treatment, with microfiltration, activated carbon filtration, and UV disinfection being the three most commonly employed treatment methods, and with many systems incorporating multiple treatment steps (Table A in S1 File, [9]).

Sierra Leone’s Ministry of Health and Sanitation (MoHS), as well as non-governmental organizations (NGOs) working in the country, are concerned about potential health risks from PSW products of unknown quality [9]. Sierra Leone has legislation regulating packaged water, and the Sierra Leone Standards Bureau (SLSB) established national standards for packaged water quality in 2010; however, the regulatory framework is uncoordinated, with unclear roles among government agencies, and standards are not effectively enforced (*idem*). Implementing and enforcing regulations is complicated by the transient nature of small PSW producers, who can easily relocate and rebrand to avoid regulatory sanctions.

Basic data on PSW quality in SL could inform government efforts to refine and enforce packaged water regulations, and to implement monitoring and surveillance efforts (*idem*). However, government agencies in SL lack such data, nor have prior studies systematically examined PSW in that country. In this work, we assessed the quality of PSW manufactured and sold in Freetown, SL, (where most of SL’s PSW producers are concentrated). To place these findings in context, we simultaneously assessed the quality of household water used for consumption (HWC) in a representative sample of households. To our knowledge, this is one of the first studies to assess and compare the chemical and microbiological quality of PSW products and HWC in a sub-Saharan African context, and the first such study to incorporate random sampling, appropriate analytical methods, and quality assurance/quality control (QA/QC) procedures [21]. This work provides insights into the safety of an increasingly important source of water for consumption in SL and West Africa, and will inform policymakers seeking to regulate and monitor PSW in SL and elsewhere, while ensuring progressive realization of access to safe water.

## Methods

### 2.1 Sampling procedure

Packaged water manufacturing facilities (PWMFs) within SL were enumerated using records from Sierra Leone’s Packaged Water Taskforce—an inter-ministerial body working to coordinate the regulation of the industry. Local markets and retail shops were also visited to identify additional PW brands not included in the database, and the contact information printed on the packaging of these brands was used to update the PWMF database. The majority of identified PWMFs (83/117) were located in Freetown (Table B in S1 File). Further sampling activities focused on producers in the capital, as this provided a convenient sample that included the majority of the country’s PW industry. A random sample of 49 Freetown PWMFs was selected; Since the PW industry in Freetown (and this sample) are dominated by PSW producers, PWMFs primarily producing bottled water (n = 5) were excluded from the analysis, leaving 44 PWMFs that primarily or exclusively produced sachets. Retail shops and street vendors in Freetown (25 each) were also selected for point-of-sale (POS) sample collection: Freetown was divided into quadrants, and a main thoroughfare with a high density of PW shops and vendors was identified in each. Enumerators walked down each thoroughfare from one end to the other, sampling each third store on alternating sides of the street. A total of 25 stores were sampled in this manner. The same approach was then used to sample 25 street vendors on thoroughfares with a high density of such vendors. At each selected POS, enumerators recorded GPS coordinates, identified all brands of packaged water for sale, and sampled one brand at random (by drawing numbers out of a bag). POS samples comprising BW (n = 4) were excluded, leaving 46 PSW samples.

A random sample of 60 households was also enumerated. Briefly, Freetown was divided into 10 sections of approximately equal area, and a main street was identified within each. Enumerators walked down the street, sampling each third house on alternating sides of the street until six households were surveyed in each section, for a total of 60. At each household, respondents were asked to provide a glass of the water that they used for drinking. A brief questionnaire was also administered, and self-reported data were collected on drinking water collection and storage practices, the source of the collected water sample (e.g. piped supply, dug well, or protected spring), as well as the storage conditions (e.g. stored vs. continuous in-house supply, method of serving if stored), and the type of household water treatment method used, if any (S2 File). Oral informed consent was obtained from all household survey participants and documented by enumerator’s written certification that consent was obtained. The decision to use oral consent was based on the rationale that because literacy rates in Sierra Leone are low [22], asking respondents to sign a written consent form would be unethical, since many potential respondents would be unable to understand it. Furthermore, it was anticipated that restricting the study to the small minority of respondents able to read and understand a written consent form would bias the sample towards those with higher levels of education and wealth, and would consequently invalidate any comparison of the health risks associated with packaged water and water from other sources, particularly for Freetown’s more vulnerable populations. This work was reviewed and received a formal waiver from the Institutional Review Board at The University of North Carolina at Chapel Hill (Study # 13–2165). The IRB found the full study exempt, including the use of oral consent; no additional separate waiver was given for the use of oral consent or any other specific aspect of the study. No identifiable personal information was collected from any participants.

### 2.2 Sample collection

PSW samples were collected at three stages in the manufacturing and distribution chain (Table C in S1 File): 1) Raw influent water (Raw samples); and 2) Finished PSW products (PWMF samples) were sampled at each PWMF; 3) PSW products were sampled at the POS (POS samples). Each raw water sample comprised three 300-mL sample aliquots collected in either sterile 500-mL glass bottles or sterile 710-mL Whirl-pak bags (Nasco, Fort Atkinson, WI). These aliquots included one unamended aliquot for all chemical analyses except nitrate and arsenic, one acidified aliquot (3 mL x 1 M HCl per 300-mL aliquot) for nitrate and arsenic analyses, and one unamended aliquot for microbiological analysis. Finished PW samples were placed in secondary plastic bags, except for aliquots to be sampled for arsenic and nitrate, which were preserved by acidification, as above. All POS samples were purchased prior to collection and analysis. The exterior surfaces of sachets were sampled at the POS (“exterior sample”) as follows: one enumerator held the PSW product with sterile gloves while a second enumerator rinsed the exterior with 300 mL of sterile buffer. The rinsate was collected in sterile Whirl-pak bags. A glass of household drinking water was received from each respondent by an enumerator and poured into sterile Whirl-Pak bag.

### 2.3 QA/QC

Quality assurance/quality control procedures included the daily collection of field blanks and duplicate samples (at least 10% of all samples, each). All field and lab blanks were free from detectable *E*. *coli* and TC; 62% and 62% of *E*. *coli* and TC duplicates had relative deviations of <25%, respectively; 94% and 69% of *E*. *coli* and TC duplicates had absolute deviations of < = 5 colony-forming units (CFUs)/100mL, respectively. Upon collection, all samples were immediately placed on ice and transported to the laboratory in coolers at 1–4°C (verified using WarmMark temperature indicators, Shockwatch, Dallas, TX). Samples were refrigerated at 4°C upon arrival at the laboratory, and analyzed within 12 hours of collection (longer holding times were due to logistical constraints, although most samples were analyzed within 6 hours). Previous research shows that such holding times have little effect on measured *E*. *coli* concentrations at holding temperatures below 10°C, although analysis should always be conducted as rapidly as possible [23]. The SLSB laboratory in Freetown, Sierra Leone carried out all chemical and microbiological analyses.

### 2.4 Laboratory analyses

Physico-chemical parameters measured were: pH, conductivity, and free chlorine (measured on-site during sample collection); turbidity, total hardness, fluoride, iron, manganese, nitrate, and arsenic (measured at SLSB). Nitrate and arsenic analyses were performed using acidified aliquots (3 mL x 1 N HCl per 300-mL aliquot), while all other analyses were performed using non-acidified aliquots. These methods are summarized in Table D (in S1 File).

Sachets for microbiological analysis were aseptically opened in the SLSB laboratory using ethanol-cleaned scissors. Samples were analyzed for *E*. *coli* and total coliforms (TC) via membrane filtration [24]. A 100-mL sample was filtered through a 0.45 μm membrane (Millipore, Billerica, MA). The filters were then placed on RAPID’ *E*. *coli* 2 Agar (Bio-Rad, Hercules, CA) plates and incubated at 35°C for 24 hours.

### 2.5 Data analysis

Results were analyzed using Stata/IC 13 (Statacorp, College Station, TX). Wilcoxon signed-rank tests were used to compare log concentrations of *E*. *coli* and TC between raw water and finished PSW samples from PWMFs and to compare PSW from the POS and exterior samples. Wilcoxon rank-sum (Mann-Whitney) tests were used to compare log concentrations of *E*. *coli* and TC between samples from various sampling points. McNemar’s test (for paired data) and Fisher’s exact test (for unpaired data) were used to compare the proportion of positive samples from various sampling points and conditions.

Samples producing colonies that were too numerous to count (TNTC) were reported as the highest countable concentration of *E*. *coli* or TC (250 CFU/100 mL). For the purpose of calculating log EC and TC concentrations, values of 0.5 CFU/100 ml were substituted for those samples in which no CFUs were detected. Adjusted geometric mean concentrations were calculated using these adjustments. While PSW samples at the POS were collected in duplicate, the results of the first replicate POS sample were always used for hypothesis-testing and quantitative comparisons (sensitivity analysis found no significant differences in the results between the two replicates). Statistical significance for all hypothesis tests was assessed at the 5% and 1% levels.

## Results

### 3.1 Microbiological water quality

#### 3.1.1 Raw water

**Fig 1:**
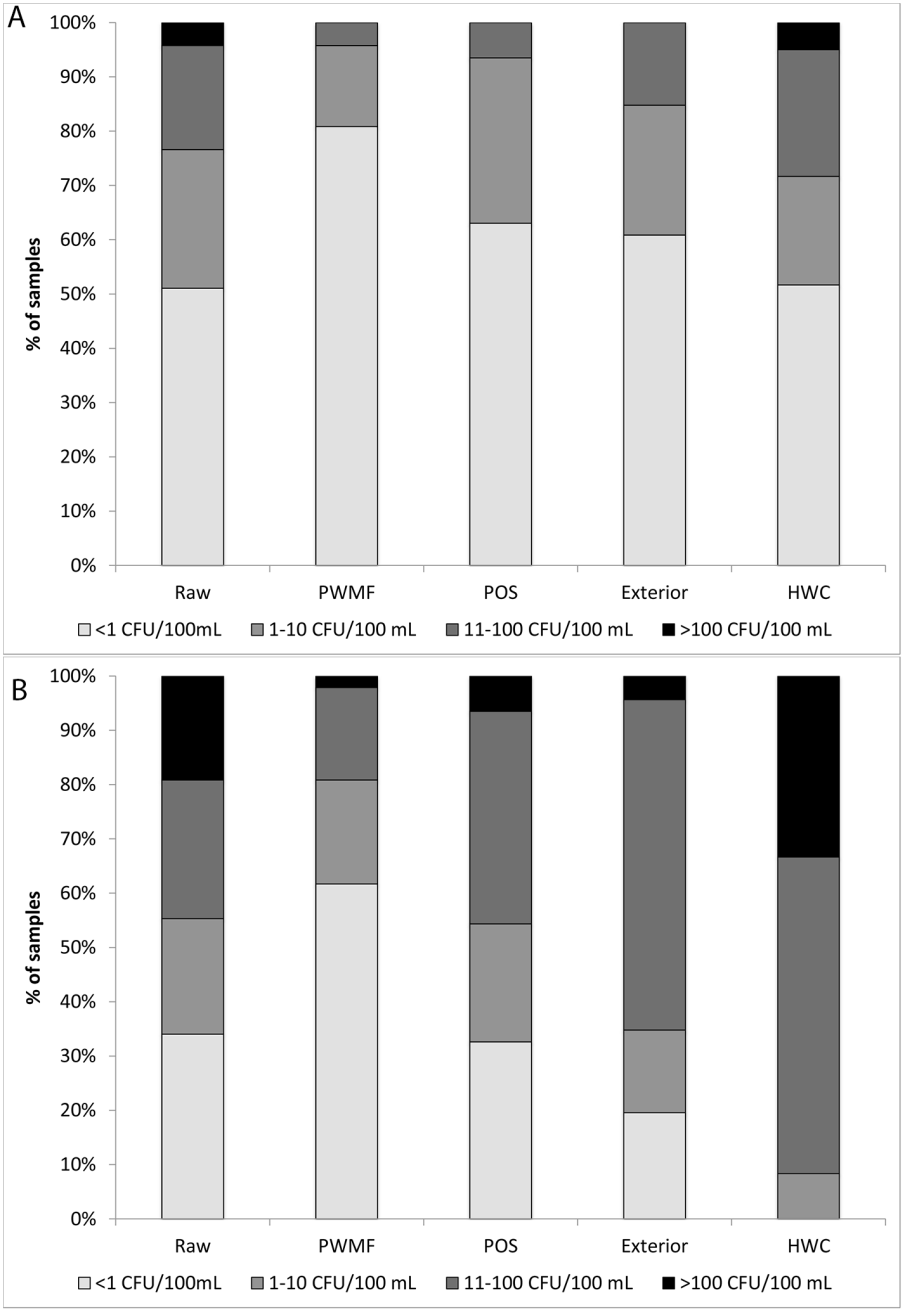
Bacterial concentrations in samples by risk category. Concentrations of A) *E*. *coli* and B) Total coliforms (CFU/100 mL) measured in raw water and finished packaged sachet water (PSW) samples at the packaged water manufacturing facility (“Raw” and “PWMF”, respectively) and in PSW samples as well as on the exterior of PSW samples at the point of sale (“POS” and “Exterior”, respectively) and in household water for consumption (“HWC”).

**Fig 2:**
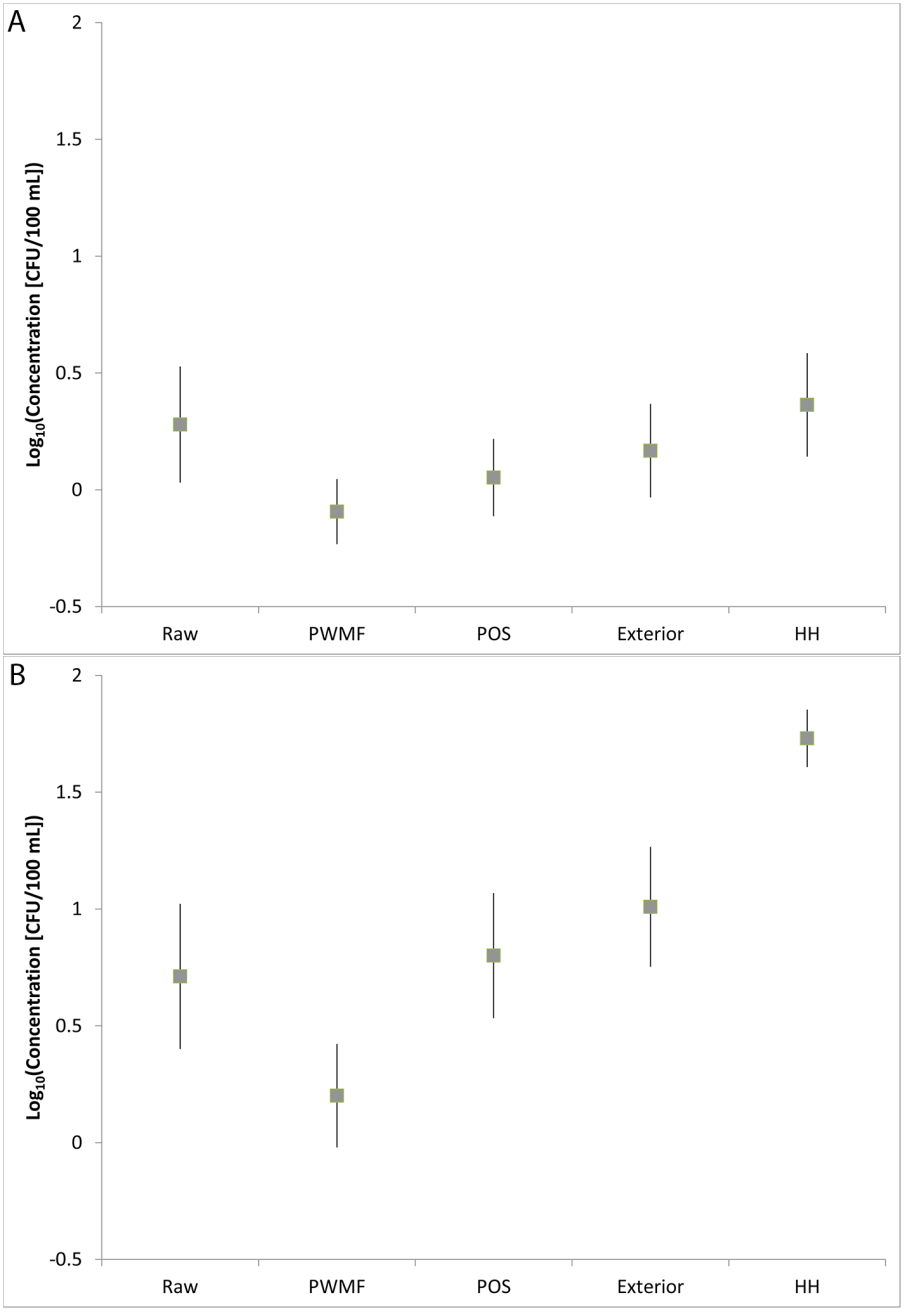
Log bacterial concentrations in samples. Log of A) *E*. *coli* and B) Total coliform concentrations (CFU/100 mL) measured in raw water and finished packaged sachet water (PSW) samples at the packaged water manufacturing facility (“Raw” and “PWMF”, respectively) and in PSW samples as well as on the exterior of PSW samples at the point of sale (“POS” and “Exterior”, respectively) and in household water for consumption (“HWC”). Boxes represent average log concentrations, whiskers represent 95% confidence intervals for log concentrations.

Of raw water samples at the PWMF, 49% contained one or more *E*. *coli* CFU/100 mL, while 66% contained detectable TC (Table 1, Fig 1). A substantive fraction of samples contained >10 CFU/100 mL *E*. *coli* (23%) and TC (45%), but few samples contained >100 CFU/mL (4% and 19%, respectively). Adjusted geometric mean concentrations were 2.2 and 5.8 CFU/mL for *E*. *coli* and TC, respectively (Table 1, Fig 2).

**Table 1:** Microbial results for raw, finished packaged sachet water (PSW) samples, and household stored water samples.

Risk Level (WHO, 1997)	CFU/100 mL	Raw water n = 47		Finished PW (PWMF) n = 47		Finished PW (POS) n = 46		Exterior Samples n = 46		HH Water for Consumption n = 60	
		*E*. *coli*	TC	*E*. *coli*	TC	*E*. *coli*	TC	*E*. *coli*	TC	*E*. *coli*	TC
Conformity	<1	51% (24)	34%(16)	81% (38)	62%(29)	63% (29)	33% (15)	61% (28)	20% (9)	52% (31)	0% (0)
Low	1–10	26% (12)	21%(10)	15% (7)	19%(9)	30% (14)	22% (10)	24% (11)	15% (7)	20% (12)	8% (5)
Intermediate	11–100	19% (9)	26%(12)	4% (2)	17%(8)	7% (3)	39% (18)	15% (7)	61% (28)	23% (14)	58% (35)
High	>100	4% (2)	19%(9)	0% (0)	2%(1)	0% (0)	7% (3)	0% (0)	4% (2)	5% (3)	33% (20)
Adjusted Geometric mean (95% CI)		2.17 (1.22–3.86)	5.75 (2.88–11.48)	-0.78 (0.58–1.05)	1.48 (0.91–2.39)	1.15 (0.79–1.66)	6.10 (3.35–11.12)	1.48 (0.95–2.30)	10.34 (5.88–18.22)	2.31 (1.40–3.83)	53.13 (40.25–70.14)

#### 3.1.2 Manufacturing facility

Most finished PSW samples at the PWMF were free from detectable *E*. *coli* and TC (81% and 62%, respectively; Table 1, Fig 1), and few samples contained >10 *E*. *coli* (4%) or TC (19%) CFU/100 mL, while fewer contained > 100 CFU/100 mL (0% and 2%, respectively). Finished PSW products at the PWMF had significantly lower log concentrations of *E*. *coli* and TC than raw water samples (p<0.01) and were significantly less likely to contain detectable *E*. *coli* and TC than raw water samples (p<0.01, Table 2).

**Table 2:** P-values for Wilcoxon-Mann-Whitney tests (unpaired data) and Wilcoxon signed-ranks tests (paired data) comparing log concentrations of fecal indicator bacteria (FIB) in samples (first row) and hypothesis tests comparing proportions of positive samples (second row) (McNemar’s chi square test for paired data, and Fisher’s exact for unpaired data) for samples collected at different points in the supply chain.

	Finished PSW (PWMF)		Finished PSW (POS)		Exterior PSW		HH Water for Consumption	
	*E*. *coli*	TC	*E*. *coli*	TC	*E*. *coli*	TC	*E*. *coli*	TC
**Raw water**	0.0037**	0.0006**	0.1231	0.9688	---	---	0.8845	0.0000**
	0.0009**	0.0001**	0.388	1.000	---	---	1.00	0.000**
**PSW PWMF**	---	---	0.0723	0.0008**	---	---	0.0009**	0.0000**
			0.160	0.31*	---	---	0.002**	0.000**
**PSW POS**	---	---	---	---	0.5148	0.1190	0.0757	0.0000**
	---	---	---	---	0.212	0.127	0.323	0.000**
**Exterior PSW**	---	---	---	---	---	---	---	---
	---	---	---	---	---	---	---	---

*indicates statistically significant difference at 95%.

**indicates statistically significant difference at 99%.

When PWMF samples were disaggregated by producer characteristics, a non-significant trend was observed towards lower frequency and concentrations of *E*. *coli* and TC for the largest and smallest 20% of producers (by total production volume) vs. producers with production volumes in the middle three quintiles, as well as for producers using a disinfection method (chlorine, ozone, ultraviolet [UV], or reverse osmosis) vs. those not using disinfection, and for those with a license to produce PSW vs. those without a license (Table E in S1 File).

#### 3.1.3 Point of Sale

46 PSW samples representing 32 brands were randomly obtained at the POS. Of these samples, 25 were collected from street vendors and 21 from retail shops. The majority of finished PSW samples at the POS (63%) had *E*. *coli* concentrations <1 CFU/100 mL, while 33% were free from detectable TC (Table 1, Fig 1). There was no significant difference in the proportion of samples containing detectable *E*. *coli* or TC, or in the log concentrations of *E*. *coli* or TC, between samples from street vendors vs. retail shops (Table F in S1 File). However, exterior samples collected from street vendors were significantly more likely to contain detectable *E*. *coli* and TC than exterior samples from retail shops, and had significantly higher concentrations of both types of indicators (Table F in S1 File).

The microbiological quality of drinking water improved significantly with treatment, and TC concentrations increased significantly along the finished PSW supply chain (i.e., from PWMF to POS), while there was a nonsignificant trend towards increasing *E*. *coli* concentrations along the supply chain (p<0.001 and p = 0.07, respectively). Finished PSW products at the POS were not significantly more or less likely to contain detectable *E*. *coli* or TC than finished PW samples or raw (influent) water samples obtained from PWMFs, nor did POS samples contain significantly different log concentrations of *E*. *coli* from PWMF or raw water samples; however, TC concentrations in POS samples were significantly higher than those of finished PW samples at the PWMF, although they were not significantly different from raw water TC concentrations (Table 2, Fig 2).

#### 3.1.4 Household water samples

When asked for a glass of the water they used for drinking, respondents provided samples from household storage containers in all cases except one, which was collected directly from an in-home tap. The origin of water stored in household containers was primarily the piped municipal water supply serving most of Freetown (62%), with the remaining households using protected dug wells (21%), protected springs (10%), and unprotected springs (7%, Table G in S1 File). 85% of users reported that they consume PW when at home.

*E*. *coli* were detected in 52% of HWC samples, while 28% and 5% of samples contained >10 and >100 *E*. *coli* CFU/100 mL, respectively. TC were detected in all HWC samples, with 92% and 33% containing >10 and >100 TC CFU/100 mL, respectively (Table 1, Fig 1). The adjusted geometric mean concentrations of *E*. *coli* and TC for HWC were 2.3 and 53.1 CFU/100 mL, respectively. The presence of *E*. *coli* in household samples was not correlated with the original source of the sample, household water treatment, or the method of extracting water from the storage container (Table H in S1 File). No comparisons were made for TC, since all samples were positive.

The log concentration values for *E*. *coli* and TC in HWC were significantly higher than in finished PSW samples from the PWMF, and significantly higher than TC values for PW samples from the POS; there was a nonsignificant trend towards higher *E*. *coli* concentrations in HWC than in PW at the POS (p = 0.08; Table 2, Fig 2). The proportion of samples with detectable *E*. *coli* was not significantly different between household water for Consumption (HWC) samples and POS samples, although HWC samples were significantly more likely to contain detectable TC than POS samples. By contrast, HWC samples were significantly more likely to contain detectable *E*. *coli* and TC than PSW samples collected at the PWMF (Table 2, Fig 2). The proportion of samples with *E*. *coli* and TC concentrations >10 CFU/100 mL were both greater for HWC samples than PSW samples collected at the PWMF and POS (p<0.01, P<0.001, respectively)).

HWC samples had significantly higher log concentrations of TC, but not *E*. *coli*, than raw water samples at the PWMF (Table 2, Fig 2). HWC samples were also significantly more likely to contain detectable TC, but not *E*. *coli*, than raw water samples collected at the PWMF. A significantly higher proportion of HWC samples also contained TC (but not *E*. *coli*) concentrations >10 CFU/100 mL relative to raw water samples collected at the PWMF (Fisher exact test, p = 0.000, p = 0.49, respectively).

#### 3.1.5 Bottled water samples

Due to the small number of BW samples obtained at the PWMF and POS, these samples were excluded from the current study. However, it is interesting to note that all BW samples were free from *E*. *coli* and TC at the PWMF (n = 5), all BW samples were free from *E*. *coli* at the POS (n = 4), and all but one sample was free from TC at the POS (Table I in S1 File). BW samples were not significantly less likely to contain *E*. *coli* or TC (p>0.05), nor did they have significantly lower concentrations of either indicator than PSW samples (p>0.05), presumably owing to the small number of BW samples obtained in our random sampling.

### 3.2 Physico-chemical water quality

The majority of raw water samples and PSW samples collected at both the PWMF and POS were in compliance with national and WHO guidelines for eight of the physico-chemical parameters measured, but not for pH and manganese (Table 3). A minority of raw and PSW samples at the PWMF fell outside the specified pH range of 6.5–8.5, as did over half of POS samples, while the majority of all samples exceeded manganese limits of 0.4 mg/L (Table 3). However, deviations from standards were not large, with all samples containing <0.7 mg/L Mn and falling within the pH range 4.5–8.0. No significant changes in physico-chemical water quality were observed along the supply chain; PW samples at the POS and PWMF resembled raw water for all physic-chemical parameters except for turbidity, which was lower for PSW than for raw water.

**Table 3:** Results of chemical analysis for raw water and finished packaged sachet water (PSW) samples. Mean (range), % compliant with WHO guidelines, (n).

Parameter	National Standard (SLSB, 2010)	WHO Guideline [26]	Raw water	Finished PSW (PWMF)	Finished PSW (POS)	HH Water for Consumption
			n = 47	n = 47	n = 46	n = 60
pH	6.5–8.5	6.5–8.5	6.58 (5.14–7.64)66% (31)	6.57 (5.01–7.81) 64% (30)	6.31 (4.84–7.75) 48% (22)	5.64 (3.95–6.90) 3% (2)
Total hardness	500 mg/L	500 mg/L	5 (2–10) 100% (46)	5 (2–10) 100% (46)	6 (2–14) 100% (46)	11 (2–40) 100% (60)
TDS	1000 mg/L	1000 mg/L	6 (2–9) 100% (47)	6 (2–9) 100% (47)	7 (0–13) 100% (46)	24 (5–132) 100% (60)
Free chlorine	N/A)	0.5 mg/L	0.1 (0.1–0.2) 100% (47)	0.1 (0.1–0.2) 100% (47)	0.1 (0.1) 100% (46)	0.1 (0–0.1) 100% (60)
Turbidity	5 NTU	5 NTU	1.12 (0.16–7.91) 98% (47)	0.50 (0.11–3.12) 100% (46)	0.54 (0.17–0.96) 100% (46)	1.14 (0.17–3.64) 100% (60)
Fluoride	1.5 mg/L	1.5 mg/L	ND^a^ 100% (47)	ND 100% (47)	ND 100% (46)	0.025 (0–0.3) 100% (60)
Iron	300 μg/L	300 μg/L	12.1 (8.2–17.3) 100% (47)	12.0 (7.2–19.7) 100% (47)	10.7 (1.4–16.4) 100% (46)	20.0 (13.5–32.8) 100% (60)
Manganese	400 μg/L	400 μg/L	463 (240–590) 6% (5)	450 (5–651) 10% (3)	418 (43–590) 26% (11)	451 (327–600) 27% (16)
Arsenic	10 μg/L	10 μg/L	ND 100% (47)	ND 100% (47)	ND 100% (46)	ND 100% (60)
Nitrate	50 mg/L	50 mg/L	5 (3–7) 100% (47)	4 (2–6) 100% (47)	4 (2–6) 100% (46)	3 (2–5) 100% (60)

^a^ ND—not detect.

The majority of HWC samples were in compliance with national standards and WHO guidelines for all physico-chemical parameters measured except pH and manganese (Table 3). Most household water samples (97%) fell outside the national standards for pH and manganese, with values ranging from pH 4.0–6.9 and 0.3–0.6 mg/L, respectively (Table 3).

## Discussion

### 4.1 Water quality of packaged water products

The results suggest that the majority of PSW products manufactured in Freetown conformed with national and international guidelines for *E*. *coli* at the time that they were produced, and did not contain chemical contaminants at concentrations posing a substantive risk to human health.

#### 4.1.1 Physico-chemical quality

Other PW studies have also reported pH values below the WHO recommended range in Sri Lanka, [25] and above the recommended range in Nigeria [15]. The WHO guidelines for pH (6.5–8.5) are aimed at reducing corrosion in metal pipes [26], and thus may not be relevant to PSW; however, use of acidic municipal piped water for manufacturing PSW products may correspond to heightened risk of contamination from lead, copper, and other metals that can leach from distribution pipes, and these should be periodically monitored by local water utilities and by PWMFs using municipal water supplies.

Other studies have also reported high manganese concentrations in PW; A recent Nigerian study found 42.5% of bottled and sachet samples exceeded 0.4 mg/L, with concentrations as high as 12.9 mg/L [27]. While chronic exposure to high manganese concentrations has been associated with neurological and cognitive impairment, the concentrations reported in this study (< = 0.6 mg/L) probably do not represent a substantial increase in risk relative to the Tolerable Daily Intake of 0.06 mg/kg of body weight established by the WHO [28].

#### 4.1.2 Microbiological quality

The finding that a minority of PSW products sold in Freetown, SL do not meet applicable standards of microbiological water safety is consistent with reports of contamination in PSW products from other West African countries. Addo et al. [29] detected *E*. *coli* and TC in 6.7% and 83.3% of samples obtained from a Nigerian PWMF, respectively (n = 30), although another Nigerian study found no detectable *E*. *coli* in 30 samples analyzed [11], and a recent study in Accra, Ghana, found similar results [30]. Variable results across studies are to be expected: reported concentrations of fecal indicator bacteria (FIB) in PSW may vary with raw water quality, treatment methods and efficacy, sampling locations and methods, as well as with the analytical media and methods used. Furthermore, few previous PSW studies in SSA have reported using QA/QC protocols or cold chain management. Thus, while microbiological results should be compared across PSW studies with great caution, it is useful to review the current findings in the context of other studies of PSW in SSA, many of which report detecting *E*. *coli* and/or TC in PSW samples.

The observed reduction in log concentration of *E*. *coli* and TC between raw water and finished PSW products in this study is consistent with the finding that all PSW producers reported treating their influent water prior to packaging. However, the presence of detectable *E*. *coli* and TC, as well as turbidity levels as high as 3 NTU, in some finished PSW samples suggests that some producers may not be adhering to best practices and/or that their treatment processes may be insufficient.

Furthermore, other contaminants beyond those tested in this study, including additional microorganisms (e.g. bacteria, human enteric viruses, and parasites), chemical contaminants, and/or radionuclides, could be present in finished PSW products sold in Freetown. Studies of PW in other settings have detected a variety of bacteria [15, 31], protozoan cysts [31], and other parasites, fungi, and molds [32–35]. These findings suggest that many organisms, particularly more recalcitrant contaminants such as protozoan cysts, helminth eggs, and fungi, may be common in raw water used for PW production, and may pass intact through some treatment processes used in PW manufacturing.

The current study obtained only a small number of BW samples, and the prevalence and concentrations of EC and TC in these samples were not significantly different from those in PSW samples; however, the data are suggestive of a trend towards better microbiological quality in BW vs PSW in Freetown. This trend merits further study with a larger sample size to determine what manufacturing and/or distribution process variables may account for any differences in safety. Since the cost per liter of BW may be substantially higher than that of PSW, substitution of BW for PSW may not be an option for many LMIC residents, but improving the safety of PSW through improvements in policy and practice may be feasible.

### 4.2 Changes in water quality along PSW supply chains

The greater TC (but not *E*. *coli*) concentrations found in samples at the POS vs. the PWMF may be due to growth of microorganisms already present within PSW products and/or to recovery of damaged microorganisms rendered viable but non-culturable (VBNC) by treatment processes (exogenous contamination seems unlikely, as sachets are hermetically sealed and are transported in secondary packaging).

Dada et al. [11] also found significantly greater prevalence of detectable TC in PSW samples from shops (40%) and street vendors (45%) vs PWMFs (6.7%) in Nigeria. However, as in our current study, Dada et al. did not find a significant difference in TC contamination between sachets from retail stores and those from street vendors. In contrast, a study from Rio de Janeiro, Brazil found greater concentrations of TC, fecal coliforms, and *Pseudomonas aeruginosa* in bottled water samples obtained from street vendors vs. commercial establishments [36].

Ejechi and Ejechi [37] found higher prevalence of detectable TCs and fecal coliforms on the exteriors of sachets from street vendors (100% and 47%, respectively) vs. retail stores (45% and 6%, respectively), consistent with our findings for *E*. *coli* and TC. Egwari et al. [38] detected *E*. *coli* on the exteriors of 29% of sachet samples (n = 96) obtained from POSs in Lagos, Nigeria, but did not detect *E*. *coli* in the contents of any samples. They also found greater TC contamination in PW products obtained from pails and wheelbarrows compared to samples obtained from refrigerators. While we found no difference in TC concentrations between PW from street vendors and retail shops, perhaps due to the small sample size, our finding that TC increased along the supply chain are consistent with the implication of El-Salam et al. (2008) that TC concentrations increase in PW stored at ambient temperatures [31].

The study team observed that PSW products were frequently transported, stored, and/or sold at ambient temperature conditions, and this may be a factor in the observed increase of TC concentrations along the supply chain. Further work investigating the effects of storage and transport conditions, time between production and sale, organic carbon content of PSW, and other factors on TC concentrations at the POS may be of interest. However, it is useful to recall that growth of TC does not necessarily indicate an increase in the concentrations of infectious human pathogens in PSW.

### 4.3 Comparison of PW to alternative sources

HWC was not significantly different from PSW with respect to the physico-chemical parameters studied. However, HWC samples had significantly higher prevalence and concentrations of *E*. *coli* and TC than PSW samples collected at the PWMF, and significantly higher TC concentrations than PSW collected at the POS, with a strong (nonsignificant) trend towards higher *E*. *coli* concentrations in HWC vs PSW at the POS as well. Thus, while improvements are needed in the microbiological safety of PSW in Freetown, PSW may be safer than the drinking water stored in many homes. With only 60% of Sierra Leone’s population having access to improved sources of drinking water [39], and with many improved sources also providing contaminated water [21], many consumers may resort to alternative sources, including PSW, surface water, and open wells; among these options, PSW may be the safest. Table J (in S1 File) summarizes selected prior studies comparing the microbiological quality of drinking water sources and PW products; many of these studies support our conclusion that, while improvements in the microbiological safety of PW products is needed, PSW may be a safer alternative to stored water from municipal and private water supplies in some LMIC settings.

### 4.4 Policy implications

The results of this work have important policy implications for PSW regulation in SL and other LMICs where PSW consumption is substantial. The prevalence of microbiological contamination in PSW products and the deterioration of water quality along the supply chain suggest the need to improve PSW manufacturing, transportation, and storage practices. Increased regulatory oversight may support these improvements [40], and should emphasize microbiological safety relative to the physico-chemical parameters included in this study. Such oversight should include monitoring, surveillance, training and education of producers, distributors, and retailers, and provisions for identification and removal of problematic PSW products from the market. Despite the potential risks associated with PSW products in SL, regulators should to avoid dissuading consumers from drinking PSW, as it may often be safer than alternative sources of HWC. While PSW may be an important source of water for consumption, it cannot replace other sources for the majority of domestic needs such as cooking, bathing, etc., as this would be impractical from logistical and economic perspectives. While the sample size in this study did not provide conclusive results for BW, BW may prove safer than PSW, and its use should not be discouraged; however, BW consumption volumes may be insignificant compared to PSW consumption in Freetown, and regulatory efforts should thus focus on improving the safety of PSW, while not discouraging its use, to maximize health gains.

Despite the large and growing importance of PSW [9], monitoring programs have been slow to collect data on this drinking water source. For example, sachet water was only introduced in the most recent MICS for Ghana [4], despite the importance of PSW in this country over the last decade. Monitoring of PSW use and safety by governments and development agencies will be an important step in regulating its production and distribution. However, as policymakers work to regulate PSW, they should also improve the safety and reliability of HWC by improving municipal drinking water supplies and expanding continuous access to these supplies. Additionally, they should assess the potential of safe household water storage and/or treatment for those households that currently lack such access in their home.

### 4.5 Limitations of this study

While useful indicators of microbiological water quality, FIB they have important limitations: FIB are more sensitive to chemical and UV disinfection than some recalcitrant pathogens [41];TC can multiply in water at ambient temperatures [42]; and FIB rendered VBNC by treatment processes may recover under some conditions [43]. Finally, TC have many non-fecal environmental sources [34], and *E*. *coli* has non-fecal sources as well [25, 27]. Nevertheless, most plausible pathways for contamination of PSW by FIB imply increased risk of fecal contamination, suggesting that differences in the prevalence and concentration of *E*. *coli* among water types in this study indicate differences in microbiological safety, while differences in TC prevalence and concentration in PSW samples at the PWMF indicate differences in treatment efficiency. While FIB are imperfect indicators, their regular use in PSW monitoring and quality control would represent a substantial improvement over current practice.

The current work comprised a moderate-sized cross-sectional study; a larger longitudinal study would facilitate robust comparison of PSW safety across subgroups (shops vs. street vendors, producer size, treatment methods, BW vs. PSW, etc.), and across batches and seasons. Furthermore, the use of more robust sampling techniques (i.e. enumerating and randomizing all vendors and households in Freetown; a method that was cost-prohibitive for the current study) could better prevent bias towards shops and households readily accessible from the main street.

### 4.6 Next steps

Further work should assess the concentrations of more recalcitrant microorganisms (viruses, protozoan cysts, etc.) as well as additional chemical contaminants (heavy metals, radioisotopes) in PSW products, piped water, and HWC in Freetown and other LMIC settings to more thoroughly assessing the potential health risks to PSW consumers.

Further exploration of the mechanisms by which water quality deteriorates along the supply chain may also be of interest, with potential implications for regulatory policy and monitoring. More broadly, Policy research is needed on best practices for safe manufacturing and distribution of PSW products in LMIC settings. Such work could emphasize cost-effective approaches to regulating and monitoring an industry in which manufacturers materialize and disappear or re-brand overnight [5]. Efforts to improve physico-chemical water may focus on collaboration with municipal utilities to ensure proper monitoring, treatment, and distribution of water throughout service areas. Such policies should be developed and implemented in a manner that takes into account the safety of both PSW and other sources of water for consumption, as addressing either in isolation may lead to unintended adverse consequences.

## Conclusions

While many sachet water products sampled in Freetown, Sierra Leone were free from *E*. *coli* and TC, some PSW products contained levels of *E*. *coli* corresponding to intermediate human health risk levels. There is a clear need for improved manufacturing process controls, as well as enhanced monitoring and regulation of PSW products manufactured and sold in SL to improve their safety. Nevertheless, comparisons with HWC samples suggest that sachet water may be safer than many alternative sources of water for consumption currently used by Freetown residents. Water and health sector stakeholders should not dissuade consumers from consuming PSW until the safety of alternate sources can be ensured, and increasing in-home access to safe and reliable municipal drinking water in Freetown should remain a top priority. Until such infrastructure improvements are realized, PSW may remain the least unsafe option for many households.

## Supporting Information

S1 File Supporting Information Tables. Table A. Percentage of producers reporting the use of various water treatment methods. Table B. Geographic distribution of all producers in Freetown compared to distribution of all surveyed producers nationwide. Table C. Types and numbers of aliquots collected for chemical and microbiological water quality testing. Table D. Methods of analysis, equipment, and detection limits for different physical and chemical parameters. Table E. Microbiological quality of PSW samples from PWMF vs. producer characteristics. Table F. Microbiological quality of PSW samples collected from street vendors vs. retail shops: P-values for rank-sum tests comparing log concentrations of fecal indicator bacteria in samples (first row) and Fisher’s exact test comparing proportions of positive samples (second row) collected from street vendors vs. retail shops. Results shown for both sachet contents (PSW) and sachet exteriors (Exterior). Table G. Household drinking water sources. Table H. Microbiological quality of stored water samples vs. household storage conditions. Table I. Microbiological quality of bottled water samples. Table J. Selected studies comparing microbiological quality of packaged water products with other drinking water sources.
(DOCX)

S2 File Supporting Information Survey Instruments. This file contains the survey instruments used for data collection in this study. The file contains a packaged water manufacturing facility (PWMF) questionnaire as well as a household questionnaire.
(DOCX)

S3 File Supporting Information Raw Data. This file contains all raw data related to the work presented in this study. The file contains all data used in the analyses presented in this study, as well as additional data, collected through the administration of the questionnaires in S2 File, that were not included in the scope of this study. All identifiable information has been anonymized to prevent disclosing the identities of household survey respondents and PWMF survey respondents.
(XLSX)

S4 File Supporting Information POS List. This file contains a list of points of sale (POSs) visited in this study. POSs are listed by POS ID, and GPS coordinates for each POS are included. POS IDs beginning with “store” correspond to retail shops, while POS IDs beginning with “street” correspond to street vendors and hawkers.
(XLSX)

## References

[1] Prüss A, Kay D, Fewtrell L, Bartram J. Estimating the burden of disease from water, sanitation, and hygiene at a global level. Environmental Health Perspectives. 2002;110(5):537–42. 12003760 10.1289/ehp.110-1240845 PMC1240845

[2] Beverage Marketing Corporation. 2011 The Global Bottled Water Market. 2012.

[3] Stoler J, Weeks JR, Otoo RA. Drinking water in transition: a multilevel cross-sectional analysis of sachet water consumption in Accra. PLOS One. 2013;8(6):e67257 23840643 10.1371/journal.pone.0067257 PMC3686721

[4] Ghana Statistical Service. Ghana Multiple Indicator Cluster Survey with an Enhanced Malaria Module and Biomarker, 2011, Final Report. Accra, Ghana: 2011.

[5] Stoler J, Weeks JR, Fink G. Sachet drinking water in Ghana’s Accra-Tema metropolitan area: Past, present, and future. Journal of Water, Sanitation, and Hygiene for Development. 2012;2(4). 10.2166/washdev.2012.104 24294481 PMC3842094

[6] Opatunji O, Obhiambo F. Consumption practices and user perception of an emerging alternative drinking water option (sachet water) in Ibadan, Nigeria. 35th WEDC International Conference; Loughborough, UK.2011.

[7] Dada AC. Packaged water: optimizing local processes for sustainable water delivery in developing nations. Global Health. 2011;10:1744–8603. 10.1186/1744-8603-7-24 PMC3161851 21801391

[8] Vapnek J, Jusu-Sheriff Y, Williams AR, Jalloh MB, Jalloh MF, Saquee G, et al Improving the Regulation, Monitoring, and Quality of the Packaged (Sachet and Bottled) Water Industry in Sierra Leone; and Sensitising the Customer Base Legislative review and recommendations. Chapel Hill, NC, USA and Freetown, Sierra Leone: University of North Carolina at Chapel Hill and FOCUS 1000, 2014.

[9] Williams AR, Jalloh MB, Jalloh MF, Saquee G, Pratt S, Fisher M, et al Improving the regulation, monitoring, and quality of the packaged (sachet and bottled) water industry in Sierra Leone; and sensitising the customer base-Final Report. Chapel Hill, NC, USA and Freetown, Sierra Leone: The University of North Carolina at Chapel Hill and FOCUS 1000, 2014.

[10] Oluwafemi F, Oluwole ME. Microbiological Examination of Sachet Water Due to a Cholera Outbreak in Ibadan, Nigeria. Open Journal of Medical Microbiology. 2012;2:115.

[11] Dada A. Sachet water phenomenon in Nigeria: Assessment of the potential health impacts. African Journal of Microbiology Research. 2009;3(1):015–21.

[12] Kwakye-Nuako G, Borketey P, Mensah-Attipoe I, Asmah R, Ayeh-Kumi P. Sachet drinking water in Accra: the potential threats of transmission of enteric pathogenic protozoan organisms. Ghana medical journal. 2007;41(2). 10.4314/gmj.v41i2.55303 PMC1976297 17925844

[13] Obiri-Danso K, Okore-Hanson A, Jones K. The microbiological quality of drinking water sold on the streets in Kumasi, Ghana. Letters in Applied Microbiology. 2003;37(4):334–9. 12969499 10.1046/j.1472-765x.2003.01403.x

[14] Ackah M, Anim A, Gyamfi E, Acquah J, Nyarko E, Kpattah L, et al Assessment of the quality of sachet water consumed in urban townships of Ghana using physico-chemical indicators: A preliminary study. Advances in Applied Science Research. 2012;3(4).

[15] Ajayi A, Sridhar M, Adekunle L, Oluwande P. Quality of packaged waters sold in Ibadan, Nigeria. African Journal of Biomedical Research. 2008;11(3).

[16] Orisakwe OE, Igwilo IO, Afonne OJ, Maduabuchi J-MU, Obi E, Nduka JC. Heavy metal hazards of sachet water in Nigeria. Archives of environmental & occupational health. 2006;61(5):209–13. 17891889 10.3200/AEOH.61.5.209-213

[17] Ajayi O, Adesida G. Radioactivity in some sachet drinking water samples produced in Nigeria. Iranian Journal of Radiation Research. 2009;7(3):151–8.

[18] Mintz ED, Reiff FM, Tauxe RV. Safe water treatment and storage in the home: A practical new strategy to prevent waterborne disease. Journal of the American Medical Association. 1995;273(12):948–53. 10.1001/jama.1995.03520360062040 7884954

[19] Shields K, Bain R, Cronk R, Wright JA, Bartram J. Influence of supply type on relative quality of source and stored drinking water in developing countries: a bivariate meta-analysis. Environmental Health Perspectives. In submission. 10.1289/ehp.1409002 PMC4671240 25956006

[20] Hunter PR, Zmirou-Navier D, Hartemann P. Estimating the impact on health of poor reliability of drinking water interventions in developing countries. Science of the total environment. 2009;407(8):2621–4. 10.1016/j.scitotenv.2009.01.018 19193396

[21] Bain R, Cronk R, Wright J, Yang H, Slaymaker T, Bartram J. Fecal contamination of drinking-water in low and middle-income countries: a systematic review and meta-analysis. PLOS Medicine. 2014;11(5). 10.1371/journal.pmed.1001644 PMC4011876 24800926

[22] UNICEF. Sierra Leone Multiple Indicator Cluster Survey 2010. 2010.

[23] Pope ML, Bussen M, Feige MA, Shadix L, Gonder S, Rodgers C, et al Assessment of the Effects of Holding Time and Temperature on Escherichia coli Densities in Surface Water Samples. Applied and Environmental Microbiology. 2003;69(10):6201–7. 10.1128/aem.69.10.6201-6207.2003 14532081 PMC201187

[24] Oshiro R. Method 1604: Total Coliforms and Escherichia coli in water by membrane filtration using a simultaneous detection technique (MI Medium). Washington, DC: US Environmental Protection Agency 2002.

[25] Herath A, Abayasekara C, Chandrajith R, Adikaram N. Temporal variation of microbiological and chemical quality of noncarbonated bottled drinking water sold in Sri Lanka. Journal of food science. 2012;77(3):M160–M4. 10.1111/j.1750-3841.2011.02588.x 22384963

[26] WHO. Guidelines for Drinking-water quality, 4th Ed Geneva, Switzerland: 2011.

[27] Alhassan MM, Ujoh F. Assessment of the Chemical Quality of Potable Water Sources in Abuja, Nigeria. British Journal of Applied Science & Technology. 2012;2(2):146–72.

[28] WHO. Manganese in Drinking-water Background document for development of WHO Guidelines for Drinking-water Quality. Geneva, Switzerland: 2011.

[29] Addo K, Mensah G, Bekoe M, Bonsu C, Akyeh M. Bacteriological quality of sachet water produced and sold in Teshie-Nungua suburbs of Accra, Ghana. African Journal of Food, Agriculture, Nutrition and Development. 2009;9(4).

[30] Stoler J, Tutu RA, Ahmed H, Frimpong LA, Bello M. Sachet Water Quality and Brand Reputation in Two Low-Income Urban Communities in Greater Accra, Ghana. The American journal of tropical medicine and hygiene. 2014;90(2):272–8. 10.4269/ajtmh.13-0461 24379244 PMC3919231

[31] El-Salam A, Al-Ghitany E, Kassem M. Quality of bottled water brands in Egypt part II: Biological water examination. The Journal of the Egyptian Public Health Association. 2008;83:468–86. 19493513

[32] Alli J, Okonko I, Alabi O, Odu N, Kolade A, Nwanze JC, et al Parasitological evaluation of some vended sachet water in Southwestern Nigeria. New York Science Journal. 2011;4(10).

[33] Cabral D, Fernández Pinto VE. Fungal spoilage of bottled mineral water. International Journal of Food Microbiology. 2002;72(1):73–6. 11843415 10.1016/s0168-1605(01)00628-6

[34] Fujikawa H, Wauke T, Kusunoki J, Noguchi Y, Takahashi Y, Ohta K, et al Contamination of Microbial Foreign Bodies in Bottled Mineral Water in Tokyo, Japan. Journal of applied microbiology. 1997;82(3):287–91. 12455891 10.1046/j.1365-2672.1997.00353.x

[35] Osei AS, Newman MJ, Mingle J, Ayeh-Kumi PF, Kwasi MO. Microbiological quality of packaged water sold in Accra, Ghana. Food Control. 2013;31(1):172–5.

[36] Nunes F, Sérgio A, Sant'Ana AS, Cruz AG. Commercialization Conditions and Practices Influence the Microbiological Quality of Mineral Waters. Journal of Food Protection®. 2008;71(6):1253–7. 18592755 10.4315/0362-028x-71.6.1253

[37] Ejechi E, Ejechi B. Safe drinking water and satisfaction with environmental quality of life in some oil and gas industry impacted cities of Nigeria. Social Indicators Research. 2008;85(2):211–22.

[38] Egwari L, Iwuanyanwu I, Ojelabi C, Uzochukwu O, Effiok W. Bacteriology of sachet water sold in Lagos, Nigeria. East African Medical Journal. 2005;82(5). 10.4314/eamj.v82i5.9312 16119752

[39] Stoler J, Fink G, Weeks JR, Otoo RA, Ampofo JA, Hill AG. When urban taps run dry: Sachet water consumption and health effects in low income neighborhoods of Accra, Ghana. Health & Place. 2012;18(2):250–62. 22018970 10.1016/j.healthplace.2011.09.020 PMC3274644

[40] Vantarakis A, Smaili M, Detorakis I, Vantarakis G, Papapetropoulou M. Diachronic long-term surveillance of bacteriological quality of bottled water in Greece (1995–2010). Food Control. 2013;33(1):63–7.

[41] Barrell R, Hunter P, Nichols G. Microbiological standards for water and their relationship to health risk. Communicable disease and public health/PHLS. 2000;3(1):8 10743312

[42] LeChevallier MW. Coliform regrowth in drinking water: a review. Journal-American Water Works Association. 1990;82(11):74–86.

[43] McFeters G, Kippin J, LeChevallier M. Injured coliforms in drinking water. Applied and Environmental Microbiology. 1986;51(1):1–5. 3513698 10.1128/aem.51.1.1-5.1986 PMC238806

