# Supplementary material for: Microbiological and Chemical Quality of Packaged Sachet Water and Household Stored Drinking Water in Freetown, Sierra Leone
Source: PLoS One. 2015 Jul 10;10(7):e0131772. doi: 10.1371/journal.pone.0131772 (PMC4498897; doi:10.1371/journal.pone.0131772)
Supplement: S1 File — Table A. Percentage of producers reporting the use of various water treatment methods. Table B. Geographic distribution of all producers in Freetown compared to distribution of all surveyed producers nationwide. Table C. Types and numbers of aliquots collected for chemical and microbiological water quality testing. Table D. Methods of analysis, equipment, and detection limits for different physical and chemical parameters. Table E. Microbiological quality of PSW samples from PWMF vs. producer characteristics. Table F. Microbiological quality of PSW samples collected from street vendors vs. retail shops: P-values for rank-sum tests comparing log concentrations of fecal indicator bacteria in samples (first row) and Fisher’s exact test comparing proportions of positive samples (second row) collected from street vendors vs. retail shops. Results shown for both sachet contents (PSW) and sachet exteriors (Exterior). Table G. Household drinking water sources. Table H. Microbiological quality of stored water samples vs. household storage conditions. Table I. Microbiological quality of bottled water samples. Table J. Selected studies comparing microbiological quality of packaged water products with other drinking water sources. (DOCX) [file pone.0131772.s001.docx]

Microbiological and chemical quality of packaged sachet water and household stored drinking water in Freetown, Sierra Leone

Michael B. Fisher^a^*^†^; Ashley R. Williams^a^*; Mohamed F. Jalloh^b^; George Saquee^b^; Robert E. S. Bain^a, c^; Jamie K. Bartram^a†^

*MBF and ARW are co-primary authors and contributed equally to this work.

^a^Department of Environmental Sciences and Engineering, University of North Carolina, Chapel Hill, NC USA

^b^FOCUS 1000, Freetown, Sierra Leone

^c^UNICEF, New York, NY, USA.

^†^Corresponding Authors: Address correspondence to either author [mbfisher@gmail.com](mailto:mbfisher@gmail.com) (MBF); [jbartram@email.unc.edu](mailto:jbartram@email.unc.edu) (JKB)

S1 File Supporting Information Tables

Supporting Information Table A. Percentage of producers reporting the use various water treatment methods.

| **Treatment Method** | **% Producers^a^** |
| --- | --- |
| Rapid sand | 16% |
| Slow sand | 26% |
| Microfiltration | 87% |
| Activated Carbon | 30% |
| Reverse osmosis | 5% |
| Coagulation | 1% |
| Chlorination | 17% |
| Ozone | 1% |
| Ultraviolet disinfection | 49% |
| Ion Exchange | 31% |
| PP sediment filter | 8% |

^a^Total >100% as several producers use multiple treatment processes.

Supporting Information Table B. Geographic distribution of all producers in Freetown compared to distribution of all surveyed producers nationwide.

|  | **Freetown Producers** | | **Surveyed Producers** | |
| --- | --- | --- | --- | --- |
| **Zone** | No. | Proportion | No. | Proportion |
| East | 32 | 39% | 19 | 39% |
| Central | 10 | 12% | 5 | 10% |
| West | 33 | 40% | 20 | 41% |
| W. Rural | 8 | 10% | 5 | 10% |
| **Total** | **83** | **100%** | **49** | **100%** |

Supporting Information Table C. Types and numbers of aliquots collected for chemical and microbiological water quality testing.

| **Sample Type** | **Description** | **No. of replicates collected** | |
| --- | --- | --- | --- |
|  |  | **Chemical** | **Microbiological** |
| Raw Water | Sample collected from source within production site, immediately before entering any treatment process | 2 | 1 |
| Packaged Water (PWMF) | Sample collected immediately as product comes off production line | 2 | 1 |
| Point of Sale (POS) | Sample collected from packaged water at POS | 2 | 2 |
| Exterior | Sample collected from exterior surface of packaged water at POS | 0 | 1 |
| Household water (HH) | Sample collected from source of household water for consumption | 2 | 2 |

Supporting Information Table D. Methods of analysis, equipment, and detection limits for different physical and chemical parameters.

| **Parameter** | **Method** | **Equipment** | **Detection Limit** |
| --- | --- | --- | --- |
| pH | USEPA Method 150.1 | Hanna Instruments HI 98129 pH/TDS meter | 0-14 pH |
| Total hardness | USEPA Method 130.2 |  |  |
| Total dissolved solids | USEPA Method 120.1 | HI 98129 | 0-2000 mg/l |
| Free chlorine |  | Lovibond colorimeter 2000+ test kit (3/40A disc); DPD No. 1 tablet | 0.1 - 1.0 mg/l |
| Turbidity | Adaptation of USEPA Method 180.1 | HI 98703 Portable Turbidimeter | 0 – 10 NTU  10 – 100 NTU  100 – 1000 NTU |
| Fluoride | USEPA Method 340.2 | ExStik FL700 Fluoride meter | 0.1-10 mg/l |
| Iron | Standard Methods  3500 Fe-B Phenanthroline method | PG Instruments T80 UV/VIS Spectrophotometer | 0.0-0.4 mg/l standard curve |
| Manganese | Standard Methods  3500 Mn | PG Instruments T80 UV/VIS Spectrophotometer | 0-0.6 mg/l standard curve |
| Arsenic |  | ITS Rapid Arsenic Test Kit, Model 481396 | 0-0.17 mg/L, >0.5 mg/L |
| Nitrate |  | Lovibond colorimeter 2000+ test kit (3/142 disc); Lovibond Nitrate No.1 and No. 2 tablets | 10-100 mg/L |

Supporting Information Table E. Microbiological quality of PSW samples from PWMF vs. producer characteristics.

| **Risk Level (WHO, 1997)** | **CFU**  **/100mL** | **Size quintile 1 (production volume) n=7** | | **Size quintile 2-4 (production volume) n=20** | | **Size quintile 5 (production volume) n=5** | | **Disinfection method reported: n=22** | | | **No disinfection method reported: n=22** | | **Licensed: n=27** | | | **No License: n=6** | |
| --- | --- | --- | --- | --- | --- | --- | --- | --- | --- | --- | --- | --- | --- | --- | --- | --- | --- |
|  |  | *E. coli* | TC | *E. coli* | TC | *E. coli* | TC | *E. coli* | | TC | *E. coli* | TC | *E. coli* | | TC | *E. coli* | TC |
| Conformity | <1 | 100% (7) | 71%(5) | 75%(15) | 50%(10) | 80% (4) | 80%(4) | 86% (19) | | 68% (15) | 73% (16) | 50% (11) | 85% (23) | | 67% (18) | 67% (4) | 33% (2) |
| Low | 1-10 | 0% (0) | 29%(2) | 20%(4) | 30%(6) | 20% (1) | 0%(0) | 9% (2) | | 23% (5) | 23% (5) | 18% (4) | 11% (3) | | 22% (6) | 33% (2) | 33% (2) |
| Intermediate | 11-100 | 0% (0) | 0%(0) | 5%(1) | 15%(3) | 0% (0) | 5%(1) | 5% (1) | | 9% (2) | 5% (1) | 27% (6) | 4% (1) | | 11% (3) | 0% (0) | 17% (1) |
| High | >100 | 0% (0) | 0%(0) | 0%(0) | 5%(1) | 0% (0) | 0%(0) | 0% (0) | | 0% (0) | 0% (0) | 5% (1) | 0% (0) | | 0% (0) | 0% (0) | 17% (1) |
| Adjusted Geometric mean (95% CI) | | -.693  (-0.69/-.69) | -.297  (-1.02/0.43) | -0.067  (-0.62/.49) | 0.617  (-.18/1.42) | -.335  (-1.33/-.66) | 0.073  (-2.05/2.20) | -.336  (-.77/0.10) | -.098  (-.58/0.38) | | -.093  (-.58/.40) | .701  (-.49/1.89) | -.336  (-.71/-.03) | -.056  (-0.50/0.39) | | -.259  (-0.61/0.09) | 1.08  (-.91/3.07) |

Supporting Information Table F. Microbiological quality of PSW samples collected from street vendors vs. retail shops: P-values for rank-sum tests comparing log concentrations of fecal indicator bacteria in samples (first row) and Fisher’s exact test comparing proportions of positive samples (second row) collected from street vendors vs. retail shops. Results shown for both sachet contents (PSW) and sachet exteriors (Exterior)

|  | **Store Samples** | |
| --- | --- | --- |
| **Street Samples** | *E. coli* | TC |
| **PSW** | 0.3520  0.363 | 0.5594  0.216 |
| **Exterior** | 0.0001**  0.000** | 0.0001**  0.000** |

*indicates statistically significant difference at 95%**indicates statistically significant difference at 99%

Supporting Information Table G. Household drinking water sources

| Water Source of household water for consumption | **Original source** | **# of HH** | **% of HH** |
| --- | --- | --- | --- |
| Storage container | Piped municipal supply | 36 | 61% |
|  | Protected dug well | 12 | 20% |
|  | Protected spring | 6 | 10% |
|  | Unprotected spring | 4 | 7% |
| In-house tap | Piped municipal supply | 1 | 2% |
| Total |  | 59^a^ | 100% |

^a^ data missing from one household

Supporting Information Table H. Microbiological quality of stored water samples vs. household storage conditions.

| **Risk Level (WHO, 1997)** | **CFU**  **/100mL** | **Serve: pour, tap, ladle**  **n=25** | | **Serve: dip hand or cup**  **n=35** | | **Source: Piped**  **n=36** | | **Source: Other**  **n=22** | | **Treat water: Yes**  **n=13** | | **Treat water: No**  **n=47** | |
| --- | --- | --- | --- | --- | --- | --- | --- | --- | --- | --- | --- | --- | --- |
|  |  | *E. coli* | TC | *E. coli* | TC | *E. coli* | TC | *E. coli* | TC | *E. coli* | TC | *E. coli* | TC |
| Conformity | <1 | 56% (14) | 0%(0) | 0%(0) | 0%(0) | 53% (19) | 0% (0) | 55% (12) | 0% (0) | 46% (6) | 0% (0) | 53% (25) | 0% (0) |
| Low | 1-10 | 24% (6) | 4%(1) | 4%(1) | 11%(4) | 19% (7) | 8% (3) | 23% (5) | 9% (2) | 31% (4) | 8% (1) | 17% (8) | 9% (4) |
| Intermediate | 11-100 | 20% (5) | 64%(16) | 64%(16) | 54%(19) | 22% (8) | 56% (20) | 23% (5) | 64% (14) | 15% (2) | 46% (6) | 26% (12) | 62% (29) |
| High | >100 | 0% (0) | 32%(8) | 32%(8) | 34%(12) | 6% (2) | 36% (13) | 0% (0) | 27% (6) | 8% (1) | 46% (6) | 4% (2) | 30% (14) |
| Adjusted Geometric mean (95% CI) | | 0.389  (-0.28-1.06) | 4.02  (3.63-4.40) | 1.158  (0.43-1.89) | 3.94  (3.54-4.35) | 0.842  (0.18-1.51) | 4.079  (3.72-4.44) | 0.544  (-0.25-1.34) | 3.71  (3.25-4.18) | 0.901  (-0.31-2.11) | 4.086  (3.36-4.81) | 0.820  (0.24-1.40) | 3.941  (3.63-4.25) |

Supporting Information Table I. Microbiological quality of bottled water samples.

| **Risk Level (WHO, 1997)** | **CFU**  **/100mL** | **PWMF**  **n=5** | | **POS**  **n=4** | |
| --- | --- | --- | --- | --- | --- |
|  |  | *E. coli* | TC | *E. coli* | TC |
| Conformity | <1 | 100% (5) | 100%(5) | 100%(4) | 75%(3) |
| Low | 1-10 | 0% (0) | 0%(0) | 0%(0) | 0%(0) |
| Intermediate | 11-100 | 0% (0) | 0%(0) | 0%(0) | 25%(1) |
| High | >100 | 0% (0) | 0%(0) | 0%(0) | 0%(0) |

**Supporting Information Table J. Selected studies comparing microbiological quality of packaged water products with other drinking water sources.**

| **Study** | **Location** | **Source type (N)** | **Results** | **PW sample type (N)** | **Results** |
| --- | --- | --- | --- | --- | --- |
| Ejechi and Ejechi (2008) | Nigeria | Private borehole-taken at tap (100)  Public tap (100) | 28% TC^a^  6% FC^a^  62% TC  18% FC | Sachet (500) | 22% TC  5% FC |
| Kassenga (2007) | Tanzania | Tap water (30) | 49% TC  26% FC | Bottled (80)  Sachet (50) | 4% TC  0% FC  18% TC  8% FC |
| Korfali et al. (2009) | Lebanon | Well (150)  Municipal water (75) | 22-45%^b^ *E. coli*  0-21%^b^ *E. coli* | Bottled (100) | 56% *E. coli* |
| Machdar et al. (2013) | Ghana | Communal taps (17)  Household storage containers (120)  Communal wells (16) | 0.5 *E. coli* CFU/100mL  13 *E. coli* CFU/100mL  38 *E. coli* CFU/100mL | Sachet (20) | 0.1 Mean *E. coli* CFU/100mL |
| Mannapperuma et al. (2013) | Sri Lanka | Well (20)  Rivers / streams / lakes (27) | 100% TC  100% FC  100% TC  100% FC | Bottled (27) | 19% TC  15% FC |
| Momtaz et al. (2013) | Iran | Tap water (144) | 23.4% *E. coli*  3.6% Salmonella  2.1% Vibrio cholerae | Bottled (304) | 2.6% *E. coli*  0% Salmonella  0% Vibrio cholerae |
| Nounou et al. (2013) | Saudi Arabia | Tap water (100)  Well (100) | 11% TC  30% TC | Bottled (100) | 0% TC |
| Raji et al. 2010 | Nigeria | Tap water (56)  Borehole (24)  Well (120) | 14% *E. coli*  13% *Salmonella*  14% *Shigella*  29% *E. coli*  8% *Salmonella*  17% *Shigella*  14% *E. coli*  12% *Salmonella*  8% *Shigella* | Sachet (80) | 16% *E. coli*  9% *Salmonella*  13% *Shigella* |
| Yasin et al. 2012 | Pakistan | Borehole (42)  Tap water (25)  Filter water (21) | 52% FC  64% FC  14% FC | Bottled (20) | 10% FC |

^a^ TC= total coliforms, FC= fecal coliforms

^b^ range represents results from two different sample locations.
