# Supplementary material for: Microbiological and Chemical Quality of Packaged Sachet Water and Household Stored Drinking Water in Freetown, Sierra Leone
Source: PLoS One. 2015 Jul 10;10(7):e0131772. doi: 10.1371/journal.pone.0131772 (PMC4498897; doi:10.1371/journal.pone.0131772)
Supplement: S2 File — This file contains the survey instruments used for data collection in this study. The file contains a packaged water manufacturing facility (PWMF) questionnaire as well as a household questionnaire. (DOCX) [file pone.0131772.s002.docx]

Microbiological and chemical quality of packaged water and household stored drinking water in Freetown, Sierra Leone.

Michael B. Fisher^1^*^&^; Ashley R. Williams^1&^; Mohamed Jalloh^2^; George Saquee^2^; Robert E. S. Bain^1, #a^; Jamie K. Bartram^1*^

&MBF and ARW are co-primary authors and contributed equally to this work.

^1^The Water Institute at UNC, Department of Environmental Sciences and Engineering, University of North Carolina, Chapel Hill, NC USA

^2^FOCUS 1000, Freetown, Sierra Leone

^#a^ Current Address: United Nations Children’s Fund, New York, New York, United States of America.

^*^Corresponding Authors: Address correspondence to either author [mbfisher@gmail.com](mailto:mbfisher@gmail.com) (MBF); [jbartram@email.unc.edu](mailto:jbartram@email.unc.edu) (JKB)

**Supporting Information**

**Survey Instruments**

**Producer Survey**

**Introduction**

FOCUS 1000 in Sierra Leone and the Water Institute of the University of North Carolina, Chapel Hill, USA have been contracted by the Ministry of Health and Sanitation, Sierra Leone with funding from UK’s Department of International Development (DFID) executed by Adam Smith International to undertake the following:

- Assess the baseline situation of the packaged water industry in Sierra Leone;
- Evaluate relevant institutions responsible for monitoring the quality and safety of packaged water;
- Work with relevant ministries, Cabinet and Parliament to enact packaged water legislation;
- Strengthen the capacity of packaged water regulator(s) and producers to ensure compliance and enforcement of the packaged water legislation.

Part of this assignment is to inspect all business establishments that are currently producing sachet and bottled water in the country. The purpose of the site visits is to examine your water source(s), storage facilities, treatment and filtration systems, packaging procedures/processes, and relevant sanitary and hygienic practices of water handlers.

Along with the site inspections, we will administer a questionnaire to assess the facilities, the production capacity, the skills and qualification of the staff in your industry. All responses you provide in this questionnaire will be kept strictly confidential, and will form part of the report to government for enacting legislations and regulations for the packaged water industry in the country.

FOCUS 1000 is grateful if you could answer as many questions as possible to the best of your ability.
Thanks for your time in completing this questionnaire. Please feel free to contact [Local Contact Person] should you have any issues or concerns in completing this questionnaire:
**Email** - XXX
**Phone** – XXX

Plant ID: ____________________________________ Region: _______________________

Enumerator ID: ________________________________

Quality Control (QC) ID: __________________________

**General Information**

| 1. Name of Company: |  |
| --- | --- |
| 1. Name(s) of Proprietor/Owner(s): |  |
| 1. Address of the Company: |  |
| 1. Telephone: |  |
| 1. Email Address: |  |
| 1. Name & Designation of Respondent: | **Name ___________________________**  **Designation _______________________** |
| 1. Date Survey was conducted: (DD/MM/YY) |  |
| 1. GPS location | **LAT _________________**  **LON _________________** |

**Registration, Licensing, and Certification**

1. Is your business registered with the Registrar General’s Office?
   □ Yes (Registration number: ____________________; Registration date: __________MM/YY)
   □ No
   □ Not sure
2. What is the registered classification of your company with the Registrar General’s Office?
   □ Sole-proprietor
   □ Joint-venture/partnership
   □ Limited liability company (Ltd)
   □ Other (specify: _____________________________________________________) □ Not sure
3. Is your establishment registered with the National Revenue Authority (NRA)?

□ Yes (Registration number: ______________________;

Registration date (DD/MM/YY): _____________________)
□ No

□ Not sure

1. Is your business licensed and certified to produce packaged water in Sierra Leone?
   □ Yes (License number: ________________________; Licensed date (DD/MM/YY): ______________)
   □ No
   □ Not sure
2. If yes, which authority granted you the license to produce packaged water in Sierra Leone?
   □ Ministry of Health and Sanitation (Environmental Health Division)

□ Sierra Leone Pharmacy Board

□ Ministry of Water Resources
□ Freetown City Council
□ Ministry of Trade and Industry
□ Sierra Leone Standard Bureaux
□ Factory Inspectorate

□ Other authority (specify: _____________________________________________________)

□ Not applicable

1. How much was the licensing fee? (Le) _____________________________________________
   *Please specify the frequency of the fee*: □ One-time □ Monthly □ Yearly □ Not sure
2. Which of the following were required as part of the licensing and certification process? (Select all possible that apply)

□ Inspection of production site, machinery and equipment by the licensing/certifying authority
□ Water quality testing
□ Certification of branding and labeling information on the sachets and bottles
□ Other (specify: _________________________________________________________)

**Business Operations**

1. When did your business start producing packaged water in Sierra Leone?

MM/YY ______________ (If unsure, leave blank)

1. What type of packaged water do you produce?

□ Sachet
□ Bottled

□ Both

□ Other (specify: ______________________)

1. What volume of water do your containers hold (mL)?

Sachet: ________; _________; _________

Bottle: ________; _________; _________

1. What type of container materials is used?

□ polyethylene

□ PVC

□ PET

□ Others (specify: ______________________________________________________________)

□ Don’t Know

1. Labeling: What information is on the product labels? (Select all that apply)

□ Date of manufacture

□ Use-by date

□ Name of manufacturer

□ Location of manufacturer

□ Treatment method(s) used

□ Certifying organization

□ Other (specify: ___________________________________)

□ None of the above

1. What are the sources of your water supply? (Select all that apply)
   □ Guma Valley Water Company
   □ Borehole;

□ Large diameter water well

□ Spring Box (protected spring)

□ Unprotected spring

□ Surface water (river, lake, stream); type:___________________________

□ Tanker truck

□ Unknown

1. Is raw water stored on the premises?

□ Yes; Volume (L): __________________;

Storage container type(s): _______________________
□ No

1. If raw water is stored on the premises, are storage tanks regularly disinfected?
   □ Yes; Frequency: _____________# per year; Method: _______________________
   □ No
   □ Not sure
   □ Not applicable
2. If raw water is stored on premises, has this source been approved for human consumption?

□ Yes; Agency granting approval: __________________;

Approval documentation provided?: Yes:_____/No:_____
□ No
□ Not sure
□ Not applicable

1. If raw water is transported from off premises, how is the water transported on site?

□ piping system from source to production site
□ tanker truck
□ closed tanks or bulk containers
□ open bulk containers
□ Not sure
□ Other (specify): ___________________________________

1. Do you have water quality testing data for the water source used?

□ Yes; Frequency of testing: ______________# per year;

Copy of results provided?: Yes:_____/No:_____
□ No
□ Not sure

1. Do you administer any form of treatment to the water before packaging it for consumption?

□ Yes

□ No
□ Not sure

1. What treatment(s) do you administer to the packaged water? (select all that apply)

□ Rapid sand filtration; Depth (cm): _______________________________________

□ Slow sand filtration; Percolation rate (m/h): _______________________________

□ Microfiltration; nominal pore size (microns; list all that apply): __________________;

Filter type (select all that apply):

□ Pleated membrane cartridge

□ Wound depth-filtration cartridge

□ Other depth-filtration cartridge

□ Bag filter

□ Carbon block cartridge

□ Activated carbon filtration;

Amount of activated carbon used (kg): ___________;

Flow rate (L/min): ___________;

how often is the activated carbon material replaced (days between replacements): ___________

membrane type: _________________________

□ Reverse osmosis
□ Coagulation;

Coagulant type: ___________________

dose (mg/L): _______________________
□ Aeration;

oxygen source (air/O_2_): ___________________

dose (mg/L): _______________________

□ Chlorine;

form of chlorine: __________________

dose (mg/L): _______________________

□ Ozone;

dose (mg/L): _______________________
□ Ultraviolet disinfection; dose (mJ/cm^2^): __________________

□ Ion exchange;

Type of IE material used: __________________;

how often is the IE material replaced (days between replacements): __________________
□ Other (specify: _____________________________________________________)

1. How often do you conduct water quality tests on your packaged water?
   □ Never

□ Daily; # of times per day: ______
□ One or more times per month; # of times per month: ______
□ Less than once per month; # of times per year: ______
□ Yearly
□ Not sure
□ Other (specify: _____________________________________________________)

1. Please specify the chemical, physical and microbiological tests conducted on your packaged water:
   (*Do not read all these to the respondent, just ask them to identify what tests they run and how many samples a year. Leave blank if they do not conduct any tests*)

| **Microbiological tests conducted at your lab** | | | **Physical/Chemical tests conducted at your lab** | | |
| --- | --- | --- | --- | --- | --- |
| Test | Place “Y” if performed | Samples/year | Test | Place “Y” if performed | Samples/ year |
| *E. coli* |  |  | Antimony |  |  |
| Total coliforms |  |  | Arsenic |  |  |
| Total thermotolerant coliforms |  |  | Asbestos (fiber > 10 microns) |  |  |
| Heterotrophic Plate Count |  |  | Barium |  |  |
| *Salmonella* spp. |  |  | Beryllium |  |  |
| *Enterococcus* spp. |  |  | Bromate |  |  |
| Fecal Streptococci |  |  | Cadmium |  |  |
| *Cryptosporidium* spp. |  |  | Chlorine (as Cl2) |  |  |
| *Giardia Lamblia* |  |  | Chloramines (as Cl2) |  |  |
| Enteric Viruses |  |  | Chlorine dioxide (as ClO2) |  |  |
| *Legionella* |  |  | Chlorite |  |  |
| **Other (Please list below):** |  |  | Chromium (total) |  |  |
|  |  |  | Cyanide |  |  |
|  |  |  | Fluoride |  |  |
|  |  |  | Lead |  |  |
|  |  |  | Mercury |  |  |
|  |  |  | Nickel |  |  |
|  |  |  | Nitrate |  |  |
|  |  |  | Nitrite |  |  |
|  |  |  | Selenium |  |  |
|  |  |  | Thallium |  |  |
|  |  |  | Aluminum |  |  |
|  |  |  | Chloride |  |  |
|  |  |  | Copper |  |  |
|  |  |  | Iron |  |  |
|  |  |  | Manganese |  |  |
|  |  |  | Silver |  |  |
|  |  |  | Sulfate |  |  |
|  |  |  | Total Dissolved Solids (TDS) |  |  |
|  |  |  | Zinc |  |  |
|  |  |  | 1,1,1-Trichloroethane |  |  |
|  |  |  | 1,1,2-Trichloroethane |  |  |
|  |  |  | 1,1-Dichloroethylene |  |  |
|  |  |  | 1,2,4-Trichlorobenzene |  |  |
|  |  |  | 1,2-Dichloroethane |  |  |
|  |  |  | 1,2-Dichloropropane |  |  |
|  |  |  | Acrylamide |  |  |
|  |  |  | Cis-1,2-Dichloroethylene |  |  |
|  |  |  | Benzene |  |  |
|  |  |  | Carbon tetrachloride |  |  |
|  |  |  | trans-1,2,-Dichloroethylene |  |  |
|  |  |  | Epichlorohydrin |  |  |
|  |  |  | Ethylbenzene |  |  |
|  |  |  | Methylene chloride (Dichloromethane) |  |  |
|  |  |  | (Mono)chlorobenzene |  |  |
|  |  |  | o-Dichlorobenzene |  |  |
|  |  |  | p-Dichlorobenzene |  |  |
|  |  |  | Haloacetic Acids |  |  |
|  |  |  | Styrene |  |  |
|  |  |  | Tetrachloroethylene |  |  |
|  |  |  | Toluene |  |  |
|  |  |  | Trichloroethylene |  |  |
|  |  |  | Vinyl chloride |  |  |
|  |  |  | Xylenes (total) |  |  |
|  |  |  | Bromodichloromethane |  |  |
|  |  |  | Chlorodibromomethane |  |  |
|  |  |  | Chloroform |  |  |
|  |  |  | Bromoform |  |  |
|  |  |  | Total Trihalomethanes |  |  |
|  |  |  | Benzo(a)pyrene (PAHs) |  |  |
|  |  |  | Di(2-ethylhexyl) adipate |  |  |
|  |  |  | Di(2-ethylhexyl) phthalate |  |  |
|  |  |  | Hexachlorobenzene |  |  |
|  |  |  | Hexachlorocyclopentadiene |  |  |
|  |  |  | Total Recoverable Phenolics |  |  |
|  |  |  | 2,4,5-TP (Silvex) |  |  |
|  |  |  | 2,4-D (Dichlorophenoxy acetic acid) |  |  |
|  |  |  | Alachlor |  |  |
|  |  |  | Aldicarb |  |  |
|  |  |  | Aldicarb Sulfone |  |  |
|  |  |  | Aldicarb Sulfoxide |  |  |
|  |  |  | Atrazine |  |  |
|  |  |  | Carbofuran |  |  |
|  |  |  | Chlordane |  |  |
|  |  |  | Dalapon |  |  |
|  |  |  | 1,2-Dibromo-3-chloropropane (DBCP) |  |  |
|  |  |  | Dinoseb |  |  |
|  |  |  | Dioxin (2,3,7,8-TCDD) |  |  |
|  |  |  | Diquat |  |  |
|  |  |  | Endothall |  |  |
|  |  |  | Endrin |  |  |
|  |  |  | Ethylene dibromide |  |  |
|  |  |  | Glyphosate |  |  |
|  |  |  | Heptachlor |  |  |
|  |  |  | Heptachlor epoxide |  |  |
|  |  |  | Lindane |  |  |
|  |  |  | Methoxychlor |  |  |
|  |  |  | Oxamyl (Vydate) |  |  |
|  |  |  | Pentachlorophenol |  |  |
|  |  |  | Picloram |  |  |
|  |  |  | Polychlorinated biphenyls (PCBs) |  |  |
|  |  |  | Simazine |  |  |
|  |  |  | Toxaphene |  |  |
|  |  |  | Methyl tertiary butly ether (MTBE) |  |  |
|  |  |  | Naphthalene |  |  |
|  |  |  | 1,1,2,2-Tetrachloroethane |  |  |
|  |  |  | Gross alpha particle radioactivity |  |  |
|  |  |  | Gross beta particle and photon radioactivity |  |  |
|  |  |  | Radium 226 and Radium 228 (combined) |  |  |
|  |  |  | Uranium |  |  |
|  |  |  | Color |  |  |
|  |  |  | Turbidity |  |  |
|  |  |  | pH |  |  |
|  |  |  | Odor |  |  |
|  |  |  | **Other (Please list below):** |  |  |
|  |  |  |  |  |  |
|  |  |  |  |  |  |
|  |  |  |  |  |  |
|  |  |  |  |  |  |
|  |  |  |  |  |  |
|  |  |  |  |  |  |
|  |  |  |  |  |  |
|  |  |  |  |  |  |
|  |  |  |  |  |  |
|  |  |  |  |  |  |

1. Who conducts your water quality testing?

□ An in-house analyst/technician

(provide qualifications of your in-house analyst:______________________________)
□ Sierra Leone Standard Bureau
□ Pharmacy Board, New England
□ Water Supply Division, Tower Hill
□ Fourah Bay College Lab
□ Not applicable (if no tests are conducted)

□ Other: _____________________________

1. If you conduct water quality testing in-house, does your facility have a certified water quality analysis laboratory?

□Yes, License number: _________________________;

Licensed date: _________________
□No

□Not Applicable

1. On average what is the quantity of your daily production?

| **Sachets** | **Quantity per day** | **Bottles** | **Quantity per day** |
| --- | --- | --- | --- |
| □ 300ml |  | □ 500 ml |  |
| □ 500 ml |  | □ 1 – 1.5 L |  |
| □ 1000 ml |  | □ 2 L |  |
| □ other vol: __________ |  | □ 10 L |  |
|  |  | □ 20 L |  |
|  |  | □ other vol: __________ |  |

**Occupational Sanitation, Hygiene and Safety**

1. Do you provide a separate changing room for your staff?

□ Yes

□ No

1. How often does your staff properly wash their hands (with soap and water) before handling and packaging the water at your production site?

□ Always □ Often □ Sometimes □ Seldom □ Never

1. Do you require staff to properly wash their hands with soap and water before handling and packing the water at your production site?

□ Yes

□ No

1. How often does your staff wash their hands after using the toilet at your production site?

□ Always □ Often □ Sometimes □ Seldom □ Never

1. Do you require staff to properly wash their hands with soap and water after using the toilet at your production site?

□ Yes

□ No

1. How often does your staff wear sanitary gloves while handling and packaging water?

□ Always □ Often □ Sometimes □ Seldom □ Never

1. Do you require staff to wear sanitary gloves while handling and packaging water at your production site?

□ Yes

□ No

1. How often does your staff smoke tobacco products, consume alcohol, or use illicit drugs while at your production site?
   □ Always □ Often □ Sometimes □ Seldom □ Never
2. Do you prohibit staff from smoking tobacco products, consume alcohol or use illicit drugs while on your water packaging production site?

□ Yes
□ No

1. How frequently do you clean (and sterilize where necessary) your production equipment?

□ Never
□ Daily

□ Weekly

□ Monthly

□ Other (Specify: ___________________________)

1. How frequently do you clean (and sterilize where necessary) your storage tanks?

□ Never
□ Daily

□ Weekly

□ Monthly

□ Other (Specify: ___________________________)

1. Are staff members required to undergo a medical fitness examination during the employment process?

□ Yes
□ No

1. If yes, how frequently are employees physically examined?

□ Once, as part of the employment process

□ Monthly

□ Quarterly

□ Annually
□ Other (Specify: ___________________________)

□ Not applicable

**Staffing and Human Resources**

1. How many staff members are employed at your water packaging company?
    Indicate number: __________________
2. How many of the following categories of employees do you have?

| **Category** | **Number** |
| --- | --- |
| Possess no formal training or education |  |
| Completed secondary school |  |
| Trained in a technical area relating to packaged water production |  |
| Attained post-secondary degree from an accredited academic institution |  |

1. Do you provide any training to newly hired employees?

□ Yes
□ No

1. What specific training do you provide to newly hired employees at your water packaging business?

| **Type of Training** | **“Y” if provided** | **Number of staff trained in this aspect** |
| --- | --- | --- |
| Machine |  |  |
| Electrical |  |  |
| Sales Management |  |  |
| Distribution |  |  |
| Water Quality Testing |  |  |
| Personal Hygiene |  |  |
| Safety |  |  |
| Finance/Accounting |  |  |
| Store Keeping/Procurement |  |  |
| Specify others:  ___________________ |  |  |

1. Do you provide in-service training to current employees to strengthen their occupational capacity?
   □ Yes

□ No

**Distribution and Sales**

1. Which areas of the country you operate?
   □ National coverage
   □ Urban areas
   □ Rural areas
   □ Western Area only
   □ Selected districts (specify:__________________________________________________________)
2. What is the means of distributing your products to customers? (select all that apply)
   □ Direct distribution by wheel barrows or push-carts

□ Direct distribution by truck;

Type of truck: __________________

Number of trucks: ___________

□ Direct distribution by motorcycle;

Number of motorcycles: __________________

□ Sell to distributors who deliver product to customers by vehicle or cart;

Number of distributors: __________
□ Sell to stores or other retailers who sell product to customers;

Number of stores or retailers: _______________
□ Other (specify: ________________________________)

1. Who are your main customers?
   □ General public
   □ Small shop holders
   □ Hotels
   □ Restaurants/Bars/Super Market
   □ Large institutions (e.g., colleges, schools, mining companies)
   □ Other (specify: ________________________________)
2. Are you a member of Packaged Water Association of Sierra Leone?
   □ Yes (member since: Year ____________________)
   □ No

**Logistics**

1. How many hours per day do you have power supply at your establishment on an average per day?

Hours per day: _______________

1. How often do you have power failures on an average per day?

Times per day: _______________

1. How many hours per day do you have water supply at your establishment on an average per day?

Hours per day: _______________

1. How many times do you have no water on an average per day?

Times per day: _______________

1. How many functional computers do you have?
   ________________________
2. Do you have in-house facilities for printing, copying and scanning of documents?
   □ Yes
   □ No

**Regulating The Packaged Water Industry**

1. Are you aware of any legislations/regulations with regards to the production and sale of packaged water to the general public?
   □ Yes
   □ No
2. If yes, please list legislation(s)/regulations you are ware of:

____________________________________________________________________________________________________________________________________________________________________________________________________________________________________________________________________________________________________________________________________________________________________________________________

1. What do you think the Government should do to ensure that consumers get the best product for their money? ______________________________________________________________________________________________________________________________________________________________________________________________________________________________________________________________________________________________________________________________________________________________________________________________________________________________________________________________________________________
2. Do you think that government should provide legislation and regulation to monitor the production of packaged water in Sierra Leone to ensure that good quality packaged water is sold to the general public?
   □ Yes
   □ No
3. Please briefly explain why you think the government should or should not provide legislation and regulation for the monitoring of the packaged water industry in Sierra Leone: ____________________________________________________________________________________________________________________________________________________________________________________________________________________________________________________________________________________________________________________________________________________________________________________________________________________________________________________________________________________________________________________________________________________________________________________

**Constraints and Recommendations**

1. What are the main constraints and challenges facing your business in ensuring safety and quality of the packaged water you are producing?

__________________________________________________________________________________________________________________________________________________________________________________________________________________________________________________________________________________________________________________________________________________________________________________________________________________________________________________________________________________________________________________________________________________________________________________________

1. In your view what are the key actions to be taken to improve quality of packaged water in Sierra Leone?
   ____________________________________________________________________________________________________________________________________________________________________________________________________________________________________________________________________________________________________________________________________________________________________________________________________________________________________________________________________________________________________________________________________________________________________________________

**Site Inspection Checklist (to be completed by a trained, independent inspector)**

**Plant ID: ________________________ Region: _____________________ Date: ______________________**

**Enumerator ID:_________________________________ Quality Control: ____________________**

**GPS coordinates: LAT____________________ LONG________________________**

1. Type of water source:

□ Guma Valley Water Company
□ Borehole; depth: _______________________(feet)
□ Large diameter water well
□ Spring box (protected spring)

□ Unprotected spring

□ Surface water (river, lake, stream); type:___________________________

□ Tanker truck
□ Other (specify: _____________________________________________________)

| 1. All surfaces and floors are clean | □ Yes □ No |
| --- | --- |
| 1. Reused containers are washed and disinfected before reuse using appropriate equipment | □ Yes □ No □ N/A |
| 1. Systems for washing and disinfecting reused containers are positioned in the plant to minimize post-disinfection recontamination | □ Yes □ No □ N/A |
| 1. Water containers are transported and stored under hygienic conditions before being filled | □ Yes □ No |
| 1. Treated water is packaged in an enclosed area separate from other operations under hygienic conditions | □ Yes □ No |
| 1. Dust, dirt, microorganisms in the air, and condensation are controlled and monitored | □ Yes □ No |
| 1. Ceilings and overhead fixtures have minimal build-up of dirt and condensation that might shed on product | □ Yes □ No |
| 1. Plant design and layout allows appropriate cleaning, disinfection, and maintenance to minimize contamination | □ Yes □ No |
| 1. Plant design provides effective prevention of pest access and harborage (windows either do not open or are fitted with screens, doors are in good repair, etc.) | □ Yes □ No |
| 1. Plant is free from detectable flies and other insects/pests | □ Yes □ No |
| 1. Surfaces and materials in contact with water are non-toxic and easy to maintain and clean | □ Yes □ No |
| 1. Surfaces and materials in contact with water are clean and free of visible contamination | □ Yes □ No |
| 1. Packages of water are handled and stored under hygienic conditions | □ Yes □ No |
| 1. Water for cleaning etc. is either treated to the same standards as drinking water, or is stored and transported separately from drinking water, with no possibility of mixing or contamination | □ Yes □ No |
| 1. Facilities for disposal of production waste | □ Yes □ No |
| 1. Functional drainage system | □ Yes □ No |
| 1. Adequate lighting system | □ Yes □ No |
| 1. Evidence of domestic animals (dogs, cats, birds, etc) in the facilities | □ Yes □ No |

1. (A) Size of the water packaging production site (sq ft) ________________________

(B) Flooring:

□ Tile □ Concrete □ Wooden □ Other: _________________

C) Inner walls:

□ Tile □ Concrete □ Wooden □ Other: _________________

Siting of facility:

| 1. Is the facility sited in an area prone to flooding? | □ Yes □ No |
| --- | --- |
| 1. Is the facility location in an environmentally polluted area or surrounded by industrial activities that might pose a risk to water quality? | □ Yes □ No |

Hygienic production facilities:

Adequacy of sanitation and hygiene provisions (check all that apply)

| 1. Functional toilet facilities | □ Yes □ No |
| --- | --- |
| 1. Functional hand washing facilities, including soap | □ Yes □ No |
| 1. Visible hand washing signs and instructions in toilet area | □ Yes □ No |
| 1. Availability of sanitary gloves | □ Yes □ No |

Adequacy of ventilation and safe working conditions (check all that apply):

| 1. Adequate ventilation | □ Yes □ No |
| --- | --- |
| 1. Adequate fume extraction capability | □ Yes □ No |
| 1. Adequate storage facilities for the packaged products | □ Yes □ No |
| 1. All chemicals are properly labeled and stored | □ Yes □ No |
| 1. Fire extinguishers available, regularly tested | □ Yes □ No |
| 1. Smoke detectors and alarm system | □ Yes □ No |
| 1. Clear demarcation of “dangerous areas” | □ Yes □ No □ N/A |

Worker dress and behavior:

| 1. All workers wear proper personal protective equipment when handling chemicals | □ Yes □ No |
| --- | --- |
| 1. Workers take appropriate action when coughing or sneezing | □ Yes □ No |
| 1. The office separate from the production room? | □ Yes □ No |

Adequacy of product packaging:

| 1. Single-serving or cooler bottles are sealed with non-reusable, tamper-resistant seals | □ Yes □ No □ N/A |
| --- | --- |
| 1. Workers take appropriate action when coughing or sneezing | □ Yes □ No |

1. Equipment Inspection (Verify presence and functionality of equipment identified by respondent in survey Q28) List the major piece(s) of equipment used for treating and packaging water

| Type of Equipment | Manufacturer | Model # | Functioning |
| --- | --- | --- | --- |
|  |  |  |  |
|  |  |  |  |
|  |  |  |  |
|  |  |  |  |
|  |  |  |  |
|  |  |  |  |

1. Specific treatment processes

| Rapid Sand filtration (present)  Volume  Depth  Functionality  Condition | □ Yes, Model #____________________  □ No  ___________________________________ m^3^  ___________________________________ cm  □ Working □ Broken □ Unclear  □ Poor □ Good □ Excellent |
| --- | --- |
| Slow sand filtration (present)  Percolation rate  Functionality  Condition | □ Yes, Model #_______________________  □ No  ________________________________________m/h  □ Working □ Broken □ Unclear  □ Poor □ Good □ Excellent |
| Microfiltration (present)  Nominal pore size (list all that apply)  Filter type  Functionality  Condition of filter cartridges | □ Yes, Model #_______________________  □ No  _________________________________________(microns)  □ Pleated membrane cartridge  □ Wound depth-filtration cartridge  □ Other depth-filtration cartridge  □ Bag filter  □ Carbon block cartridge  □ Working □ Not working □ Unclear  □ Poor □ Good □ Excellent |
| Activated Carbon filtration (present)  Amount of carbon used  Flow rate  Replacement filters/carbon onsite  Functionality  Condition | □ Yes, Model #______________________  □ No  ____________________________ (kg)  ____________________________ (L/min)  □ Yes □ No  □ Working □ Broken □ Unclear  □ Poor □ Good □ Excellent |
| Reverse osmosis (present)  Functionality  Condition | □ Yes, Model #_______________________  □ No  □ Working □ Broken □ Unclear  □ Poor □ Good □ Excellent |
| Coagulation (present)  Coagulant type used  Dose  Expiration date of coagulant | □ Yes, Model #_______________________  □ No  _____________________________  _____________________________ (mg/L)  ________________ |
| Aeration (present)  Oxygen source  Dose  Functionality  Condition | □ Yes, Model #____________________  □ No  _____________________________ (air/O_2_)  _____________________________ (mg/L)  □ Working □ Broken □ Unclear  □ Poor □ Good □ Excellent |
| Chlorine (present)  Form of chlorine  Dose  Expiration date of chlorine  Functionality of dosing mechanism  Condition of chlorination supplies  q40_chlor_cond | □ Yes □ No  _____________________________  _____________________________ (mg/L)  _____________________________  □ Working □ Broken □ Unclear  □ Poor □ Good □ Excellent |
| Ozone (present)  Dose  Functionality  Condition | □ Yes, Model #___________________  □ No  _______________________________ (mg/L)  □ Working □ Broken □ Unclear  □ Poor □ Good □ Excellent |
| Ultraviolet disinfection (present)  Dose  Functionality  Condition | □ Yes, Model #___________________  □ No  _______________________________ (mJ/cm^2^)  □ Working □ Broken □ Unclear  □ Poor □ Good □ Excellent |
| Ion exchange (present)    Type of IE material used  Replacement IE material onsite  Functionality  Condition | □ Yes, Model #_____________________  □ No  _______________________________  □ Yes □ No  □ Working □ Broken □ Unclear  □ Poor □ Good □ Excellent |
| Other treatment process  Specify:_________________________________  Functionality  Condition | □ Yes, Model #_____________________  □ No  □ Working □ Broken □ Unclear  □ Poor □ Good □ Excellent |

**Household Survey**

**Instructions for Interviewers:**

Please read the prompts and questions out loud to respondents. *Sentences in italics* are instructions for the interviewer and are not meant to be read out loud to respondents. Unless otherwise indicated, select only one answer for every question. Do not read the responses to the respondent, unless otherwise indicated. Allow respondents to respond and then determine which answer best describes their response. Circle the number and text of the response that best describes the respondent’s answer. If the respondent gives a response that is similar to two answers, ask clarifying questions to determine which answer best describes the respondent’s response. If the question is somehow not applicable to the respondent, write “33” in the last blank. If a respondent does not want to answer the question, write “44” in the last blank. If a respondent does not know the answer first try to explain the question or ask clarifying questions, and if they still do not know then write “55” in the last blank.

**Introduction**

Hello, I am with FOCUS 1000, a non-governmental organization in Sierra Leone. FOCUS 1000 and the Water Institute of the University of North Carolina- Chapel Hill, USA have been contracted by the Ministry of Health and Sanitation, Sierra Leone with funding from UK’s Department of International Development (DFID) executed by Adam Smith International to help improve the packaged water industry in Sierra Leone.

Before I begin, are you 18 years or older? ***If no, ask if there is another adult in the household you can speak with.***

As part of our survey, we would like to take a sample of your regular drinking water. Is your normal drinking water available for us to sample? ***If not, for example if they normally drink packaged water and do not have it available, then thank them and go to the next household. If they normally use tap water and it is not running and they do not store water, then thank them and go to the next household.***

As part of this project, we are trying to learn more about packaged water use in Freetown. The purpose of this survey is to learn about household’s drinking water sources and their use of packaged water. The survey should take 20-30 minutes of your time. As part of the survey, we would like to take a sample of your drinking water to test its quality. All responses you provide in this questionnaire will be kept strictly confidential, and will help inform our project and improve the packaged water industry in Sierra Leone. You do not have to participate in this survey and you can decline to answer any question if you so choose. You may end the survey at any point if you so choose. Your participation in their study will not impact your drinking water service in any way, nor will you receive anything for participating in this survey. There is no risk to you or your family members in participating in this survey.

Would you like to participate in this study? ***If the respondent says yes, then continue.***

The Institutional Review Board (IRB) of the University of North Carolina has approved this survey. FOCUS 1000 is grateful for your participation in this survey and appreciates your time. If you have any questions about this survey, you may contact [Local Contact Person].
**Email** - [XXX](mailto:gsaquee@focus1000.org)
**Phone** – XXX

| Date |  |
| --- | --- |
| Enumerator |  |
| Quality Control |  |
| Household ID |  |
| Household GPS Waypoint |  |

**Drinking Water Sample Collection**

We would like to begin by taking a water sample of your main drinking water source. Can you please show me where your drinking water is? (*SLSB sample collector to actually take the sample, but interviewer will accompany the respondent and SLSB sample collector*). If their normal drinking water source is unavailable, then ask to sample their alternative source.

| 1. ***Interviewer to observe****: Where is the drinking water directly collected from*? 2. Household stored water container 3. Piped supply in the household ***Skip to Q3*** 4. Piped supply in the yard ***Skip to Q3*** 5. Bottled water ***Skip to Q3*** 6. Sachet ***Skip to Q3*** 7. Other (specify): ______________________________ ***Skip to Q3***   ________ (*If needed, put “33” or “44” or “55” here*) |  |
| --- | --- |
| 1. Where did the water in your stored water container come from? 2. Piped water from GUMA into your household 3. Piped water from SALWACO into your household 4. Piped water from GUMA into your yard 5. Piped water from SALWACO into your yard 6. Public tap/standpipe 7. Tubewell/borehole 8. Protected dug well 9. Unprotected dug well 10. Protected spring 11. Unprotected spring 12. Rainwater collection 13. Vendor delivering water to your household 14. Tanker truck 15. Surface water (stream, river, lake, pond, stream, canal) 16. Neighbour’s piped supply 17. Neighbour’s protected well 18. From multiple water sources 19. Other (specify): _________________________________________   33- Not applicable  44- Declined to answer  55- Do not know |  |

| 1. What is your main water source of drinking water at home?   Q3_mainhome   1. Piped water from GUMA into your household 2. Piped water from SALWACO into your household 3. Piped water from GUMA into your yard 4. Piped water from SALWACO into your yard 5. Public tap/standpipe 6. Tubewell/borehole 7. Protected dug well 8. Unprotected dug well 9. Protected spring 10. Unprotected spring 11. Rainwater collection 12. Bottled water 13. Water cooler (20-L water bottle/dispenser) 14. Sachet water 15. Vendor delivering water to your household 16. Tanker truck 17. Surface water (stream, river, lake, pond, canal) 18. Neighbour’s piped supply 19. Neighbour’s protected well 20. Other (specify): ________________________________________   33- Not applicable  44- Declined to answer  55- Do not know | 1. Besides your main drinking water source, what other sources do you use for drinking water when you are at home? (***Circle all that apply***)   Q4_althome   1. Piped water from GUMA into your household 2. Piped water from SALWACO into your household 3. Piped water from GUMA into your yard 4. Piped water from SALWACO into your yard 5. Public tap/standpipe 6. Tubewell/borehole 7. Protected dug well 8. Unprotected dug well 9. Protected spring 10. Unprotected spring 11. Rainwater collection 12. Bottled water 13. Water cooler (20-L water bottle/dispenser) 14. Sachet water 15. Vendor delivering water to your household 16. Tanker truck 17. Surface water (stream, river, lake, pond, canal) 18. Neighbour’s piped supply 19. Neighbour’s protected well 20. Other (specify): ________________________________________   33- Not applicable  44- Declined to answer  55- Do not know |
| --- | --- |

| 1. Do you work outside your home? 2. No ***Skip to Q7*** 3. Yes   ________ |  |
| --- | --- |

| 1. What is your main water source when you are at work?   Q6_maintravel   1. Piped water from GUMA into your household 2. Piped water from SALWACO into your household 3. Piped water from GUMA into your yard 4. Piped water from SALWACO into your yard 5. Public tap/standpipe 6. Tubewell/borehole 7. Protected dug well 8. Unprotected dug well 9. Protected spring 10. Unprotected spring 11. Rainwater collection 12. Bottled water 13. Water cooler (20-L water bottle/dispenser) 14. Sachet water 15. Vendor delivering water to your household 16. Tanker truck 17. Surface water (stream, river, lake, pond, canal) 18. Neighbour’s piped supply 19. Neighbour’s protected well 20. Other (specify): ________________________________________   33- Not applicable  44- Declined to answer  55- Do not know | 1. What is your main water source when are away from home and not at work?   Q7_mainwork   1. Piped water from GUMA into your household 2. Piped water from SALWACO into your household 3. Piped water from GUMA into your yard 4. Piped water from SALWACO into your yard 5. Public tap/standpipe 6. Tubewell/borehole 7. Protected dug well 8. Unprotected dug well 9. Protected spring 10. Unprotected spring 11. Rainwater collection 12. Bottled water 13. Water cooler (20-L water bottle/dispenser) 14. Sachet water 15. Vendor delivering water to your household 16. Tanker truck 17. Surface water (stream, river, lake, pond, canal) 18. Neighbour’s piped supply 19. Neighbour’s protected well 20. Other (specify): ________________________________________   33- Not applicable  44- Declined to answer  55- Do not know |
| --- | --- |

| 1. Do you store water at your house? This does not refer to water storage tanks on roof-tops or water storage tanks connected to a piped supply. 2. No ***Skip to Q14*** 3. Yes   ________ |  |
| --- | --- |
| 1. Why do you store water at your house? 2. Water does not always flow from the tap 3. It is more convenient than fetching water repeatedly 4. It is easier to use for washing, etc. when stored in a container 5. Other (specify): _________________________________________   ________ |  |
| 1. How often do you normally clean your water storage container? This is not referring to your any rooftop storage tanks. 2. Never 3. Less than once a week 4. About once a week 5. Two to three times a week 6. Every other day 7. About every day   ________ |  |
| 1. Can you please show me how you normally get water out of your container when you want a drink? ***Interviewer to observe:*** *How do they take water out of the water container of stored water*? 2. Directly with their unwashed hand 3. Directly with their hand, after washing it 4. By a spigot or tap on the tank 5. Using a ladle or spoon 6. By dipping the cup into the container to fill it 7. Pouring it directly of an open mouthed container 8. Other (specify): _________________________________________   ________ |  |

| 1. ***Interviewer to observe****: Where is the storage container located? If more than 1 storage container,* ***circle all that apply****.* 2. In the kitchen area 3. In the bathroom of the house 4. In another room of the house (specify) ________________________ 5. The home has one room and water is stored here (not in a kitchen area) 6. Outside the house 7. Other (specify) _________________________________________   ________ |  |
| --- | --- |
| 1. ***Interviewer to observe****: Is the storage container covered?* 2. No 3. Yes   ________ |  |
| 1. Do you do anything to your water to make it safer to drink? 2. No ***Skip to Q16*** 3. Yes   ________ |  |
| 1. What do you do normally do to your water before drinking it? (***Read all options and circle all that they mention)*** 2. Boil it 3. Add bleach or chlorine 4. Use a water filter (ceramic, sand, composite) 5. Solar disinfection 6. Other (specify): _________________________________________   ________ |  |
| 1. Do you ever drink packaged water, meaning bottled or sachet water? 2. No 3. Yes ***Skip to Q18***   ________ |  |
| 1. What is the primary reason that you do not drink packaged water? 2. Cost  ***Skip to Q24*** 3. Low water quality of packaged water ***Skip to Q24*** 4. Not available in my location ***Skip to Q24*** 5. Other (specify): ________________________ ***Skip to Q24***   ________ ***Skip to Q24*** |  |
| 1. What is the primary reason that you drink packaged water? 2. Taste 3. Better quality than other water sources available 4. Convenient to buy it where I am 5. My other drinking water options are not always available 6. Social perception, to demonstrate status or wealth 7. Cheaper than other sources 8. Other (specify): _________________________________________   ________ |  |
| 1. Where do you most frequently purchase your packaged water? 2. From a retail store 3. From a street vendor 4. Both retail stores and street vendors 5. Other (specify): _________________________________________   ________ |  |
| 1. How many bottles of water do you normally drink in one day (number)? *If they do not drink bottles write “0”*   *Numerical Response __________________________* |  |
| 1. How many water sachets do you normally drink in one day (number)? *If they do not drink sachets write “0”*   *Numerical Response __________________________* |  |
| 1. How do you normally drink water from a sachet? 2. Bite into it 3. Use a knife/scissors to cut the package 4. Other (specify): _________________________________________   ________ |  |
| 1. Do you normally clean the exterior of the sachet water in any way before drinking it? 2. No 3. Yes   ________ |  |

| **Household questions**   1. What is your relationship to the head of the household? 2. Head of household 3. Husband or wife of head of household 4. Other adult in the household   ________ |  |
| --- | --- |
| 1. How many people normally live in your house (*number*)?   *Numerical Response __________________________* |  |
| 1. How many people in your house are 5 years or younger (*number*)?   *Numerical Response _________________________* ***If the answer is “0” Skip to Q28*** |  |
| 1. In the last 14 days, did you give bottled water or sachet water to anyone in your household under 5 years old? 2. No 3. Yes   ________ |  |
| **Health outcomes**  Now I am going to ask you some questions related to your health.   1. In the last 14 days have you suffered from an ear infection? 2. No 3. Yes   ________ |  |
| 1. In the last 14 days has any child 5 years or younger in your household suffered from an ear infection? 2. No 3. Yes   33- Does not have kids 5 or under in the household  ________ |  |
| 1. In the last 14 days have you suffered from diarrhoea? 2. No 3. Yes   ________ |  |
| 1. In the last 14 days has any child 5 years or younger in your household suffered from diarrhoea? 2. No 3. Yes   33- Does not have kids 5 or under in the household  ________ |  |
| **Basic demographics**  Now I am going to ask you some questions about your household.   1. What is the highest level you have completed in education? 2. Never attended school 3. Completed Primary school 4. Completed secondary school 5. Ordinary diploma 6. Higher national diploma (HND) 7. Have a Bachelor’s Degree (4 year university) 8. Have a Masters or Doctorate 9. Technical trade or specialized training   ________ |  |
| 1. What is the primary occupation of the highest-earning member of the household? 2. No occupation 3. Farming 4. Raising livestock 5. Labour or construction 6. Selling agricultural products 7. Selling other goods 8. Teaching 9. Government employee/civil servant 10. Driver or bike rider 11. Craftsmen (carpenter, metal worker, electrician) 12. Banking/finance 13. Owns a restaurant of food stall 14. Owns a business (not a farm or restaurant) 15. Pastor or Imam or other religious position 16. Other (specify): ________________________________________   ________ |  |
| 1. Do you own or rent your house? 2. Own 3. Rent 4. Other (specify)_________________________________________   ________ |  |
| 1. How many of the following items do you own that are currently functioning? 2. Radio or cassette player How many: ____________ 3. Mobile phone How many: ____________ 4. Television How many: ____________ 5. Washing machine How many: ____________ 6. Refrigerator How many: ____________ 7. Bicycle How many: ____________ 8. Motorcycle How many: ____________ 9. Car How many: ____________ |  |
| 1. ***Interviewer to observe****: Is the respondent*: 2. Male 3. Female   ________ |  |
